# Polychotomous traits and evolution under conformity

**DOI:** 10.1073/pnas.2205914119

**Published:** 2022-09-19

**Authors:** Kaleda K. Denton, Uri Liberman, Marcus W. Feldman

**Affiliations:** ^a^Department of Biology, Stanford University, Stanford, CA 94305;; ^b^School of Mathematical Sciences, Tel Aviv University, Tel Aviv, Israel 69978

**Keywords:** conformity, polychotomous trait, stable equilibria, polymorphism, global convergence

## Abstract

Conformist and anticonformist transmission of dichotomous cultural traits (i.e., traits with two variants) have been studied both experimentally, in many species, and theoretically, with mathematical models. Signatures of types of conformity to polychotomous traits (with more than two variants; e.g., baby names and syllables in bird song) have been inferred from population-level data, but there are few models that include individual-level biases among more than two discrete variants. We generalize the standard dichotomous trait conformity model by Boyd and Richerson to incorporate n≥3 role models and m≥2 variants. Our analysis shows that in the case of n=3 role models, under anticonformity, the central polymorphic equilibrium $p^{*}=(\frac{1}{m},\ldots ,\frac{1}{m})$ is globally stable, whereas under conformity, if initially the frequencies of ℓ variants are all equal to the maximum variant frequency in the population, there is global convergence to an equilibrium in which the frequencies of these variants are all $\frac{1}{\ell }$ and all other variants are absent. With a general number *n* of role models, the same result holds with conformity, whereas under anticonformity, global convergence is not guaranteed, and there may be stable frequency cycles or chaos. If both conformity and anticonformity occur for different configurations of variants among the *n* role models, a variety of novel polymorphic equilibria may exist and be stable. Future empirical studies may use this formulation to directly quantify an individual’s level of (anti)conformist bias to a polychotomous trait.

## Significance

Polychotomous traits (i.e., traits with more than two variants) are ubiquitous in nature, from phonemes to food sources. Individuals may adopt variants nonrandomly, influenced by biases including conformity and anticonformity. Mathematical models of (anti)conformity to a dichotomous trait, with two variants, are widely accepted, but fewer conformity models incorporate more than two discrete variants, and in those that do, the level of conformity is determined by a single coefficient. We generalize standard dichotomous trait conformity models—where there may be different conformity coefficients depending on the numbers of sampled variants—to include *m* variants. Frequency dynamics, including polymorphic equilibria, stable cycles, and chaos, can differ from what is known for a trait with only two variants.

Cultural traits, such as tools, ideas, components of speech, or behaviors (1), can have different types, known as variants. Dichotomous cultural traits have two variants, such as pro vs. con opinions or the presence vs. absence of behaviors. Polychotomous cultural traits have three or more variants; examples include baby names, fashions, and components of birdsong. The transmission of dichotomous and polychotomous traits may be nonrandom, or biased, and two biases that have been widely studied are conformity and anticonformity. Conformity entails that a more common cultural variant is adopted at a rate greater than its population frequency, while under anticonformity, the adoption rate is less than its frequency (2).

Many empirical studies of conformity and anticonformity have explored dichotomous cultural traits. For example, in ref. 3, children were presented with two arrays with dots and asked which had more dots, and in ref. 4, individuals were shown two shapes rotated at different angles and asked whether they were the same or different shapes. Participants conformed to the answers of others in the mental rotation task (4), and in ref. 3, young children anticonformed to others’ choices, whereas older children conformed. In nonhuman animals, conformity to a dichotomous trait was exhibited by sticklebacks choosing between feeders (5), great tits deciding whether to push a puzzle box slider to the left or right (6, 7), and female fruit flies copulating with pink or green males (8).

In ref. 9, people were shown two lines (a dichotomous trait) or more than two lines, with a maximum of six (a polychotomous trait), and were asked which line was longest. The authors found that the strength of conformity increased as the number of lines increased. They explained (ref. 9, p. 11):

“…consider a world with only 2 [variants]—black and white shirts. The presence of black shirts at anything above 50% suggests that people are selecting black shirts above chance. However, in a world with four [variants]—black, white, green, and red shirts—black shirts need only be present above 25% to suggest selection above chance…all current models and experiments may have been underestimating the strength of the conformist bias, because there are often more than 2 [variants] in the real world.”

Thus, modeling (anti)conformity to a polychotomous trait may help empiricists adequately assess levels of conformity rather than relying on dichotomous trait models. The authors also found that the strength of conformity and the reliance on social learning increased with group size for the dichotomous but not for the polychotomous trait, perhaps because as the number of variants increases, “larger groups are required for group size to have a discernible effect” (ref. 9, p. 19).

There is some evidence for (anti)conformity to real-world polychotomous traits that have hundreds to thousands of variants. As directly measuring individual biases among a large number of variants can be difficult, many studies have inferred cultural transmission biases from signatures in population-level data. For example, in ref. 10, the turnover in baby names—i.e., the number of new names that enter the list of top names—was plotted as a function of top list size (top 5 names, top 10 names, etc.). A concave plot suggested anticonformity, while a convex plot suggested conformity or content-biased transmission (where the latter is a bias for a particular variant over another). Recent distributions of common baby names appeared to be under anticonformist bias, whereas in earlier decades, common male baby names appeared to be under conformist or content-biased transmission, although these could not be distinguished (10).

Another population-level signature of conformity is the sigmoidal curve that can appear when the probability of adopting a variant is plotted against that variant’s population frequency (2). Acerbi et al. (11) contended that a sigmoidal curve could also be observed under content bias and demonstrator bias (the latter occurs if variants are copied randomly from a particular subset of individuals in the population) and questioned whether there was “strong support for the existence of conformist transmission at all” (ref. 11, p. 6). However, Smaldino et al. (12) reran the simulations in ref. 11 with different and possibly more realistic assumptions (table 1 in ref. 12) and found that the sigmoidal curve no longer appeared under content bias, while with demonstrator bias only a slightly sigmoidal curve appeared under some conditions. They concluded that sigmoidal curves are much more likely to result from conformist transmission than content-biased or demonstrator-biased transmission. When models of conformity, content bias, and demonstrator bias were each fitted to empirical data on the distributions of syllables in swamp sparrow song (13), the distributions were most consistent with conformist bias.

**Table 1.** n=3 role models and m=3 variants

	Offspring probabilities $P(A_{i}|x)$			
Role model state, x	*A*_1_	*A*_2_	*A*_3_	P(x)
(3, 0, 0)	1	0	0	$p_{1}^{3}$
(0, 3, 0)	0	1	0	$p_{2}^{3}$
(0, 0, 3)	0	0	1	$p_{3}^{3}$
(2, 1, 0)	$\frac{2}{3}+\frac{D}{3}$	$\frac{1}{3}-\frac{D}{3}$	0	$3p_{1}^{2}p_{2}$
(2, 0, 1)	$\frac{2}{3}+\frac{D}{3}$	0	$\frac{1}{3}-\frac{D}{3}$	$3p_{1}^{2}p_{3}$
(1, 2, 0)	$\frac{1}{3}-\frac{D}{3}$	$\frac{2}{3}+\frac{D}{3}$	0	$3p_{1}p_{2}^{2}$
(1, 0, 2)	$\frac{1}{3}-\frac{D}{3}$	0	$\frac{2}{3}+\frac{D}{3}$	$3p_{1}p_{3}^{2}$
(0, 2, 1)	0	$\frac{2}{3}+\frac{D}{3}$	$\frac{1}{3}-\frac{D}{3}$	$3p_{2}^{2}p_{3}$
(0, 1, 2)	0	$\frac{1}{3}-\frac{D}{3}$	$\frac{2}{3}+\frac{D}{3}$	$3p_{2}p_{3}^{2}$
(1,1,1)	$\frac{1}{3}$	$\frac{1}{3}$	$\frac{1}{3}$	$6p_{1}p_{2}p_{3}$

Despite the prevalence of polychotomous traits, the majority of the many theoretical studies of conformity involve dichotomous traits. In refs. 1 and 2, two models of frequency-dependent transmission of a dichotomous cultural trait (with variants *A* and *B*, say) were proposed, both of which could incorporate conformity or anticonformity. In ref. 2, individuals randomly sample *n* role models from the previous generation, and there are different conformity coefficients for different samples of role models (e.g., individuals might conform more strongly if 60% of role models share a variant than if 90% do, or vice versa). Many subsequent theoretical studies involving conformity to dichotomous traits have taken *n* to be three (14–21), in which case there is a single conformity coefficient, or have adopted another formulation that also includes a single conformity coefficient (18, 22, 23). Some models have added parameters for content bias (15, 24), payoff-dependent bias (16), individual learning (14, 17–19, 21), or various forms of group selection (20, 22). In ref. 25, Boyd and Richerson’s (2) conformity models for a dichotomous trait with *n* > 3 role models and more than one conformity coefficient were reexamined and shown to exhibit dynamics that were not observed with n=3 role models, including stable asymmetric polymorphic equilibria, exact cycles in variant frequencies (if n≥5), and chaos (if n≥10). In ref. 26, further generalizations allowed conformity coefficients to vary stochastically over time.

Fewer models of conformity have incorporated a polychotomous trait. Henrich (15) argues that a polychotomous trait can be approximated by a dichotomous trait in some contexts (ref. 15, p. 994): “In the typical diffusion of an innovation, tracking only two traits is sufficient to capture the essential process: Trait 1 represents the presence of the novel trait (the “innovation”), and Trait 2 indicates the absence of the trait. If we are, for example, studying the spread of a new nitrogen fertilizer, an individual possesses Trait 1 if he or she uses the fertilizer and possesses Trait 2 if he or she does not use the fertilizer.”

However, consider the following example. Prior to the occurrence of the novel type, suppose there are two types of fertilizer, denoted by *A* and *B*, each frequency at $\frac{1}{2}$. A new type, *C*, appears in the population at a small frequency, denoted by *ε*. Considering only two variants, namely, “*C*/not *C*” as in ref. 15, would lead one to conclude that under weak anticonformity, the frequency of *C* would be $\frac{1}{2}$ at equilibrium (2, 25). However, this is incorrect because with three variants, a frequency $\frac{1}{2}$ of one variant would render it common and thus disfavored under anticonformity; instead, a frequency $\frac{1}{3}$ of all variants would be the intuitive equilibrium in this case. If instead there were conformist transmission when *C* was introduced at a low frequency *ε*, then in the dichotomous *C*/not *C* framework, the type that is not *C* would be expected to reach fixation (2, 25). However, a polychotomous conformity model is more informative because it can tell us which of *A* or *B* (or neither) will fix in the population. When *C* first appears, let the frequency of *A* become $\frac{1}{2}-\eta$ and the frequency of *B* become $\frac{1}{2}-\delta$, where *η* and *δ* are small. If η<δ, then *A* will fix; if η>δ, then *B* will fix; and if η=δ, both *A* and *B* will be favored over *C*, but neither *A* nor *B* will be favored over the other, so the frequencies of *A* and *B* will be 0.5 at equilibrium (shown in *Result 5*). Thus, a polychotomous trait model is more appropriate than a dichotomous trait model in such situations.

Among the few theoretical studies of conformity with polychotomous traits, refs. 18, 23, 27 allowed social and individual learners in variable environments to acquire one of many variants, with either one (18, 23) or more (27) variants having a fitness advantage in a given environmental state. In these studies, social learners could be conformist, with a single conformity coefficient. A continuous trait was explored in refs. 28–30, where cultural transmission was a function of a group’s mean trait value (among other variables). In refs. 29 and 30, “conformists” preferentially adopted trait values equal to or near this mean trait value. This definition of conformity differs from the definition for a discrete cultural trait: in the latter case, conformists preferentially adopt the most common variant (i.e., the modal variant).

The present study explores conformist and anticonformist transmission of a discrete and selectively neutral polychotomous cultural trait (e.g., pottery motifs, dog breeds, or baby names) in a stable environment. Rather than incorporating a single conformity coefficient, we allow (anti)conformity to vary flexibly for different samples of role models provided n≥4 role models are observed. For example, if the name “Mary” is slightly more popular than other names, an individual might conform and name a child Mary, but it would be unrealistic to expect the same level of conformity if, say, 80% of all people that the individual observed were named Mary. Our previous studies (25, 26) focused on dynamical properties of the commonly studied model (2) of a dichotomous trait, which could include multiple conformity coefficients if n≥5 role models were sampled. Here we generalize ref. 25 to incorporate an arbitrary number *m* of cultural variants with *n* role models. If *n* is taken to be 3, as is often the case in models of conformity with dichotomous traits, then there is a single conformity coefficient, and there is global convergence of the population to one of two equilibria: equal frequencies of all variants initially present in the population, under anticonformity, or equal frequencies of the variant(s) that were initially present at the maximum frequency in the population, and zero frequency for all other variants, under conformity. With a general number *n* of role models and multiple conformity coefficients, conformity entails global convergence to the same equilibrium as with *n* = 3. However, with anticonformity and *n* role models, it is possible that no equilibrium is reached, and stable frequency cycles, or chaos, may arise. If individuals can conform or anticonform depending on the configuration of sampled role models, the dynamics can be much more complex. For example, a variety of novel asymmetric polymorphic equilibria may exist and be stable.

## Model

Suppose there are *m* variants $A_{1},A_{2},\ldots ,A_{m}$ in the population. Each offspring samples *n* adults, known as role models, and observes their state $x=(x_{1},x_{2},\ldots ,x_{m})$, where *x_i_* is the number of sampled role models with variant *A_i_* for i=1,2,…,m. For example, if *n* = 10 role models were observed and 3 had variant *A*_1_, 5 had variant *A*_2_, and 2 had variant *A*_4_, then x=(3,5,0,2,0,…,0). Therefore, $0\leq x_{i}\leq n$ and $\sum_{i=1}^{m}x_{i}=n$. The number of different configurations for the possible role model states is $\left(\begin{array}{c} m-1+n\\ n \end{array}\right)$.

Let $p_{1},p_{2},\ldots ,p_{m}$ be the initial population frequencies of variants $A_{1},A_{2},\ldots ,A_{m}$, respectively, and as in ref. 2, assume that the *n* role models are randomly chosen from the population. Then the probability of the state $x=(x_{1},x_{2},\ldots ,x_{m})$ is

[1] $$P(x)=\frac{n!}{x_{1}!x_{2}!\cdots x_{m}!}p_{1}^{x_{1}}p_{2}^{x_{2}}\cdots p_{m}^{x_{m}}.$$

Given ***x***, and following ref. 2, the probability that an offspring adopts type *A_i_* can be written

[2] $$\begin{array}{ll} P(A_{i}|x) & =\frac{x_{i}}{n}+\frac{D_{i}(x)}{n} \end{array}$$

for i=1,2,…,m, where $\frac{x_{i}}{n}$ is the frequency of type *A_i_* in the sample of *n* role models and $D_{i}(x)$ is the conformity coefficient for type *A_i_*, which depends on the role model state ***x***. (Thus, if individuals were neither conformist nor anticonformist biased, i.e., $D_{i}(x)=0$, the probability of adopting type *A_i_* would equal its observed frequency.) We assume that

[3a] $$-x_{i}<D_{i}(x)<n-x_{i}\;\text{for}\;i=1,2,\ldots ,m\;\text{and}$$

[3b] $$\sum_{i=1}^{m}D_{i}(x)=0.$$

The frequencies $p_{1}\prime ,p_{2}\prime ,\ldots ,p_{m}\prime$ of the *m* types $A_{1},A_{2},\ldots ,A_{m}$, respectively, in the offspring population are then $p_{i}\prime =\sum_{x}\left[\frac{x_{i}}{n}+\frac{D_{i}(x)}{n}\right]P(x)$, which can be written as

[4] $$p_{i}\prime =p_{i}+\frac{1}{n}\sum_{x}D_{i}(x)\frac{n!}{x_{1}!x_{2}!\cdots x_{m}!}p_{1}^{x_{1}}p_{2}^{x_{2}}\cdots p_{m}^{x_{m}}$$

for i=1,2,…,m. Here $\frac{1}{n}\sum_{x}x_{i}P(x)=\frac{np_{i}}{n}=p_{i}$ by taking the expectation of the multinomial distribution. For each state $x=(x_{1},x_{2},\ldots ,x_{m})$, the vector $D(x)=(D_{1}(x),D_{2}(x),\ldots ,D_{m}(x))$ of conformity coefficients for variants $A_{1},A_{2},\ldots ,A_{m}$ satisfies [**3a**] and [**3b**]. It is also assumed that D(x) has the following properties:

P(i) If type *A_i_* is absent in the sample of role models, then offspring do not acquire *A_i_*. In other words, if *x_i_* = 0, then $D_{i}(x)=0$ for i=1,2,…,m.
P(ii) D(x) is symmetric with respect to the *m* types $A_{1},A_{2},\ldots ,A_{m}$.

Property P(ii) entails that if for the state $x=(x_{1},x_{2},\ldots ,x_{m})$ we have *x_i_* = *x_j_*, then $D_{i}(x)=D_{j}(x)$. Also, if the two states $x=(x_{1},x_{2},\ldots ,x_{m})$ and $y=(y_{1},y_{2},\ldots ,y_{m})$ have identical components but in a different order, then the corresponding vectors D(x) and D(y) have the same components in the different order. That is, if $y_{j}=x_{i}$, then $D_{j}(y)=D_{i}(x)$ for i,j=1,2,…,m. Thus, for example, the extent to which an individual (anti)conforms to variant *A*_1_ given the sample x=(5,3,2) is the same as the extent to which an individual (anti)conforms to variant *A*_2_ given the sample x=(2,5,3). In both cases, the variant of interest is present in 5 of 10 role models, and two other variants are present in 3 and 2 role models. Applying properties P(i) and P(ii), we have, for example, that

[5] $$\begin{array}{l} D(n,0,\ldots ,0)=D(0,n,0,\ldots ,0)=\cdots \\ =D(0,\ldots ,0,n)=0. \end{array}$$

The symmetry property P(ii) entails that all the D(x) in Eq. **5** are equal, and by property P(i), $D_{i}(n,0,\ldots ,0)=0$ for i=2,…,m. However, as $\sum_{i=1}^{m}D_{i}(x)=0$ for any state ***x***, we also have $D_{1}(n,0,\ldots ,0)=0$ and so D(n,0,…,0)=(0,0,…,0)=0.

Similarly, if $\frac{n}{m}$ is an integer, then $D(\frac{n}{m},\frac{n}{m},\ldots ,\frac{n}{m})=(0,0,\ldots ,0)=0.$ This follows from the symmetry property P(ii), which entails that $D_{i}(\frac{n}{m},\ldots ,\frac{n}{m})=D_{j}(\frac{n}{m},\ldots ,\frac{n}{m})$ for all i,j=1,2,…,m and the fact that $\sum_{i=1}^{m}D_{i}(\frac{n}{m},\ldots ,\frac{n}{m})=0$. It also follows in general that D(k,k,0,0,…,0)=0, D(l,l,l,0,…,0)=0, and so on if $k=\frac{n}{2},\;l=\frac{n}{3}$, etc., are integers.

We now turn to some examples of the evolutionary dynamics for the recursion system in Eq. **4**.

## n=3 Role Models and m≥3 Variants

In the simplest polychotomous trait conformity model, there are *n* = 3 role models and *m* = 3 variants. Any role model state $x=(x_{1},x_{2},x_{3})$ has $x_{1}+x_{2}+x_{3}=3$. There are 10 possible states:

[6] $$\begin{array}{ll} & (3,0,0),(0,3,0),(0,0,3),(2,1,0),(2,0,1),\\ & (1,2,0),(1,0,2),(0,2,1),(0,1,2),(1,1,1). \end{array}$$

By properties P(i) and P(ii) above, the associated conformity vectors $D(x_{1},x_{2},x_{3})=D(x)=(D_{1}(x),D_{2}(x),D_{3}(x))$ (see below Eq. **4**) are

[7] $$\begin{array}{ll} & D(3,0,0)\text{​}=\text{​}D(0,3,0)\text{​}=\text{​}D(0,0,3)=D(1,1,1)=(0,0,0) \end{array},$$

and if D(2,1,0)=(D,−D,0), then by symmetry,

[8] $$\begin{array}{ll} & D(2,1,0)=(D,-D,0),\;\;D(2,0,1)=(D,0,-D)\\ & D(1,2,0)=(-D,D,0),\;\;D(1,0,2)=(-D,0,D)\\ & D(0,2,1)=(0,D,-D),\;\;D(0,1,2)=(0,-D,D). \end{array}$$

From Eq. **2** we have

[9] $$\begin{array}{ll} & \;\text{If}\;x_{i}=0\;\text{or}\;3\;\text{then}\;P(A_{i}|x)=\frac{x_{i}}{3}\\ & \;\text{If}\;x_{i}=2\;\text{then}\;P(A_{i}|x)=\frac{2}{3}+\frac{D}{3}\\ & \;\text{If}\;x_{i}=1\;\text{then}\;P(A_{i}|x)=\frac{1}{3}-\frac{D}{3}. \end{array}$$

In this case, the 10 conformity vectors $D(x_{1},x_{2},x_{3})$ are determined by one parameter *D* and by Eq. **9**, −2<D<1. We then have the offspring probabilities in Table 1.

By Eq. **8**, for example, $D_{1}(2,1,0)=D$ and $D_{2}(2,1,0)=-D$. The recursion for *A*_1_ is

[10] $$\begin{array}{l} p_{1}\prime =p_{1}^{3}\;+\;3p_{1}^{2}p_{2}\text{​}\left(\frac{2}{3}\;+\;\frac{D}{3}\right)+\;3p_{1}^{2}p_{3}\text{​}\left(\frac{2}{3}\;+\;\frac{D}{3}\right)+\;3p_{1}p_{2}^{2}\text{​}\left(\frac{1}{3}-\frac{D}{3}\right)\\ \;\;+\;3p_{1}p_{3}^{2}\text{​}\left(\frac{1}{3}\;-\;\frac{D}{3}\right)+\;6p_{1}p_{2}p_{3}\text{​}\left(\frac{1}{3}\right)\text{​}, \end{array}$$

with similar recursions for $p_{2}\prime$ and $p_{3}\prime$. Since $p_{1}+p_{2}+p_{3}=1$, these recursions can be written as

[11] $$p_{i}\prime =p_{i}+Dp_{i}[p_{i}-(p_{1}^{2}+p_{2}^{2}+p_{3}^{2})]\;\;i=1,2,3.$$

We then have the following result concerning possible equilibria and their local stability properties.

Result 1. *For* n=3 *role models*, *m=3 variants*, *and* D≠0,

(i) *The possible equilibria are* 3 “*corners*” $c_{1}=(1,0,0)$, $c_{2}=(0,1,0)$, *and* $c_{3}=(0,0,1)$; 3 “*boundary” equilibria* $b_{1}=(0,\frac{1}{2},\frac{1}{2})$, $b_{2}=(\frac{1}{2},0,\frac{1}{2})$, *and* $b_{3}=(\frac{1}{2},\frac{1}{2},0)$; *and* 1 *central polymorphic equilibrium* $p^{*}=(\frac{1}{3},\frac{1}{3},\frac{1}{3})$.
(ii) *If* D>0, *the only locally stable equilibria are the* 3 “*corners*.” *If* D<0, *the central polymorphism is the only locally stable equilibrium*.

The proof of *Result 1* is in *SI Appendix*, section A.

When *n* = 3 and there are any number *m* of variants with m≥3, then since $x=(x_{1},x_{2},\ldots ,x_{m})$ must satisfy $\sum_{i=1}^{m}x_{i}=n=3$, there are only three types of role model states ***x*** (up to symmetry), namely, (3,0,…,0), (2,1,0,…,0), and (1,1,1,0,…,0). Now D(3,0,…,0)=D(1,1,1,0,…,0)=(0,0,…,0), and if D(2,1,0,…,0)=(D,−D,0,…,0), then again all the conformity vectors $D(x_{1},x_{2},\ldots ,x_{m})$ are determined by one parameter *D*, Eq. **9** holds, and Eq. **4** reduces to

[12] $$\begin{array}{ll} p_{i}\prime & =p_{i}+Dp_{i}\left[p_{i}-\sum_{j=1}^{m}p_{j}^{2}\right]\;\;i=1,2,\ldots ,m. \end{array}$$

As in the case of *m* = 3 variants, if D≠0, then at equilibrium, either $p_{i}=0,\;p_{i}=1$, or if $p_{i}>0$ and $p_{j}>0$ with i≠j, then *p_i_* = *p_j_*. Hence, there is a central polymorphic equilibrium $p^{*}=(\frac{1}{m},\frac{1}{m},\ldots ,\frac{1}{m})$, *m* corners $c_{1}=(1,0,\ldots ,0),\;c_{2}=(0,1,0,\ldots ,0),\ldots ,c_{m}=(0,0,\ldots ,0,1)$, and boundary equilibria $\hat{p}=({\hat{p}}_{1},{\hat{p}}_{2},\ldots ,{\hat{p}}_{m})$ where some of the ${\hat{p}}_{i}$ values are zero and all of the nonzero ${\hat{p}}_{i}$ values are equal. Local stability of the equilibria is described by the following result.

Result 2. *With n=3 role models*, m≥3 *variants*, *and* D≠0:

(i) If D>0, then all the m corners $c_{1},c_{2},\ldots ,c_{m}$ are locally stable, and the other equilibria are not stable.
(ii) If D<0, the central polymorphism is the only locally stable equilibrium.

The proof of *Result 2* is similar to that of *Result 1* and is given in *SI Appendix*, section A.

In the original model of ref. 2 with *n* = 3 role models and *m* = 2 variants, there was global convergence. This is also the case here with m≥3 and *n* = 3, which we state as follows.

Result 3. *With* n=3 *role models and* m≥3 *variants*, *if* D≠0, *then starting with* $p^{\left(0\right)}=(p_{1}^{\left(0\right)},\ldots ,p_{m}^{\left(0\right)})\neq p^{*}$, *where* $p^{*}$ *is an equilibrium*, *and* $p_{i}^{\left(0\right)}>0$ *for all* i=1,2,…,m:

(i) *If* D>0 *and initially* $p_{j}=\max_{1\leq i\leq m}p_{i}$ *is unique* (*i*.*e*., $p_{j}>p_{i}$ *for all* i≠j), *then there is global convergence to the corner* $c_{j}$, *where* $p_{j}=1$.
(ii) *If* D>0 *and initially the frequencies of* l *variants are equal to* $\max_{1\leq i\leq m}p_{i}$, *then the final frequencies of these* l *variants are* $\frac{1}{l}$, *while the final frequencies of the other variants are zero*.
(iii) *If* D<0, *then* $p^{*}=(\frac{1}{m},\frac{1}{m},\ldots ,\frac{1}{m})$ *is globally stable*.

The proof of *Result 3* is given in *SI Appendix*, section A. Table 2 summarizes these and other findings for the current model with m≥3 variants and shows how they can differ from previous results of refs. 2 and 25 concerning (anti)conformist transmission of a dichotomous trait.

**Table 2.** Population dynamics for dichotomous and polychotomous traits, assuming that initially, $p_{1},\ldots ,p_{m}>0$ and the population is not at equilibrium

Transmission	Role models	Dichotomous trait (*m* = 2 variants)	Polychotomous trait (*m* > 2 variants)
Purely conformist	*n* = 3	Global convergence to a corner.	Global convergence to an equilibrium. If initially, the frequencies of l≥1 variant(s) are equal to $\max_{1\leq i\leq m}p_{i}$, the final frequencies of these l variant(s) are $\frac{1}{l}$, while other variant(s) are lost.
Purely conformist	Any *n*	Global convergence to a corner.	Same as above.
Purely anticonformist	*n* = 3	Global convergence to $p^{*}=(\frac{1}{2},\frac{1}{2})$.	Global convergence to $p^{*}=(\frac{1}{m},\ldots ,\frac{1}{m}).$
Purely anticonformist	*n* = 4	Global convergence to $p^{*}=(\frac{1}{2},\frac{1}{2})$.	Numerical results suggest convergence to $p^{*}=(\frac{1}{m},\ldots ,\frac{1}{m})$, but this has not been proven analytically.
Purely anticonformist	*n* = 5	Convergence is not guaranteed.	Numerical results suggest convergence to $p^{*}=(\frac{1}{m},\ldots ,\frac{1}{m})$, but this has not been proven analytically.
Purely anticonformist	Any *n*	Convergence is not guaranteed.	Convergence is not guaranteed.
Conformist and anticonformist	*n* = 4	Not possible.	A variety of population dynamics are possible (e.g., *Result 4* and Fig. 1).
Conformist and anticonformist	*n* = 5	It is possible that corners and $p^{*}=(\frac{1}{2},\frac{1}{2})$ are unstable, while asymmetric polymorphic equilibria are stable or vice versa.	A variety of population dynamics are possible; e.g., asymmetric interior equilibria may be stable (Fig. 2).

## n>3 Role Models and m=3 Variants

With *n* > 3 role models, there is more than one conformity coefficient, and the dynamics become more complicated than for *n* = 3. For example, with *n* = 4 role models and *m* = 3 variants, there are two conformity coefficients that can be nonzero, one corresponding to role model state x=(3,1,0) (or by symmetry, x=(1,3,0),(0,1,3), etc.) and the other corresponding to the states x=(2,1,1), (1,2,1), and (1,1,2). Denote the former by D′ and the latter by *D*, where −3<D′<1 and −2<D<2. Then, from Eq. **4**, with i≠j≠k and i,j,k∈{1,2,3},

[13] $$\begin{array}{ll} p_{i}\prime & =p_{i}+D\prime p_{i}(1-p_{i})(2p_{i}-1)\\ & \;+3p_{i}(p_{j}^{2}p_{k}+p_{j}p_{k}^{2})\left(D\prime -\frac{1}{2}D\right)+3Dp_{i}^{2}p_{j}p_{k}. \end{array}$$

If D′≠0 and/or D≠0, equilibria include corners, (1, 0, 0), (0, 1, 0), and (0, 0, 1); boundary equilibria, $\left(\frac{1}{2},\frac{1}{2},0),\;(\frac{1}{2},0,\frac{1}{2}\right)$, and $\left(0,\frac{1}{2},\frac{1}{2}\right)$; and the symmetric internal equilibrium $\left(\frac{1}{3},\frac{1}{3},\frac{1}{3}\right)$. There are no other boundary equilibria (25), but there can be three other interior equilibria (*SI Appendix*, section A), namely

[14] $$\begin{array}{ll} (p_{1}^{*},p_{2}^{*},p_{3}^{*})= & \left(\frac{-2D\prime -3D}{2D\prime -3D},\frac{2D\prime }{2D\prime -3D},\frac{2D\prime }{2D\prime -3D}\right)\text{​},\\ & \left(\frac{2D\prime }{2D\prime -3D},\frac{-2D\prime -3D}{2D\prime -3D},\frac{2D\prime }{2D\prime -3D}\right)\text{​},\\ & \left(\frac{2D\prime }{2D\prime -3D},\frac{2D\prime }{2D\prime -3D},\frac{-2D\prime -3D}{2D\prime -3D}\right)\text{​}. \end{array}$$

If D′>0, the equilibria given by Eq. **14** are valid (i.e., $0<p_{i}^{*}<1$ for *i* = 1, 2, 3) if $0<D\prime <-\frac{3}{2}D$, where *D* < 0. If D′<0, they are valid if $-\frac{3}{2}D<D\prime <0$, where *D* > 0. The local stability properties of equilibria with *n* = 4 role models and *m* = 3 variants are given as *Result 4*:

Result 4. *With n = 4 role models*, m=3 *variants*, *and* D′,D≠0:

(i) The corners are locally stable if and only if (iff) D′>0.
(ii) Boundary equilibria such as $\left(0,\frac{1}{2},\frac{1}{2}\right)$ are locally stable in their boundaries iff D′<0 and locally stable in general iff $-\frac{3}{2}D<D\prime <0$.
(iii) The central polymorphic equilibrium $p^{*}=(\frac{1}{3},\frac{1}{3},\frac{1}{3})$ is locally stable iff $D\prime <-\frac{3}{4}D$.
(iv) The three interior equilibria given by Eq. **14** are always unstable.

**Fig. 1.**
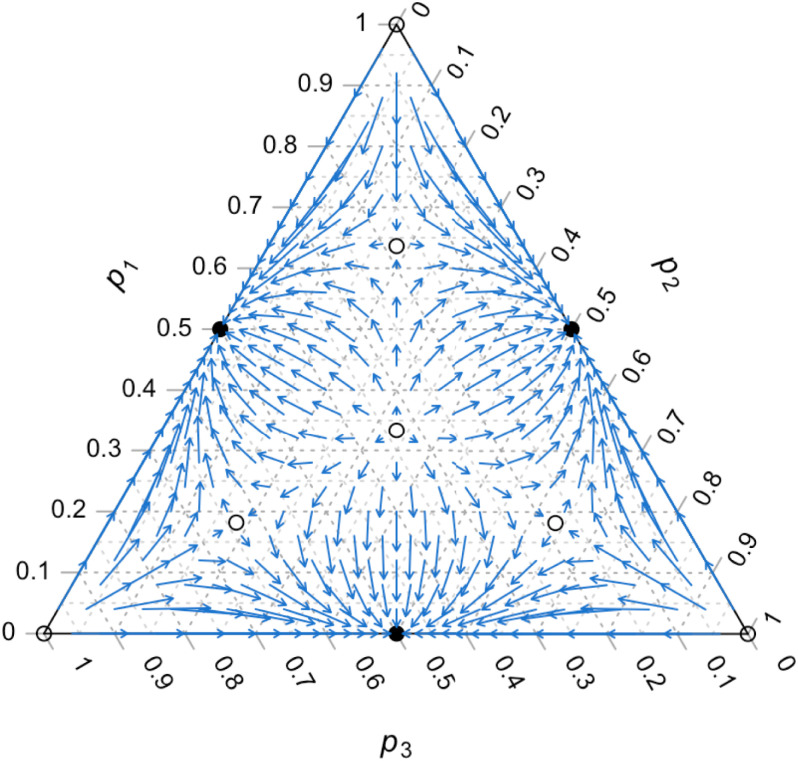
Dynamics with *n* = 4 role models and *m* = 3 variants, where D′=−0.3 and *D* = 0.9 in Eq. **13**. The base of each arrow is located at a starting position $\left(p_{1}^{\left(0\right)},p_{2}^{\left(0\right)},p_{3}^{\left(0\right)}\right)$, and the tip of the arrow is at the final position after five generations. Filled circles mark stable equilibria, and open circles mark unstable equilibria, although unstable equilibria may be stable along an axis (for example, see $p_{i}=p_{j}\neq p_{k}>0$, where i≠j≠k). Here 2D′+3D=2.1>0, so there are unstable interior equilibria given by Eq. **14**, and the equilibria $p^{*}=(\frac{1}{2},\frac{1}{2},0),\;(\frac{1}{2},0,\frac{1}{2})$, and $\left(0,\frac{1}{2},\frac{1}{2}\right)$ are stable. Corner equilibria are unstable, and $p^{*}=(\frac{1}{3},\frac{1}{3},\frac{1}{3})$ is unstable because 4D′+3D=1.5>0.

The proof of *Result 4* is in *SI Appendix*, section A. Fig. 1 illustrates a set of trajectories for the three recursions exemplified by Eq. **13**. We see that when equilibria in Eq. **14** exist, they are on curves that separate domains of attraction to the three boundary equilibria.

**Fig. 2.**
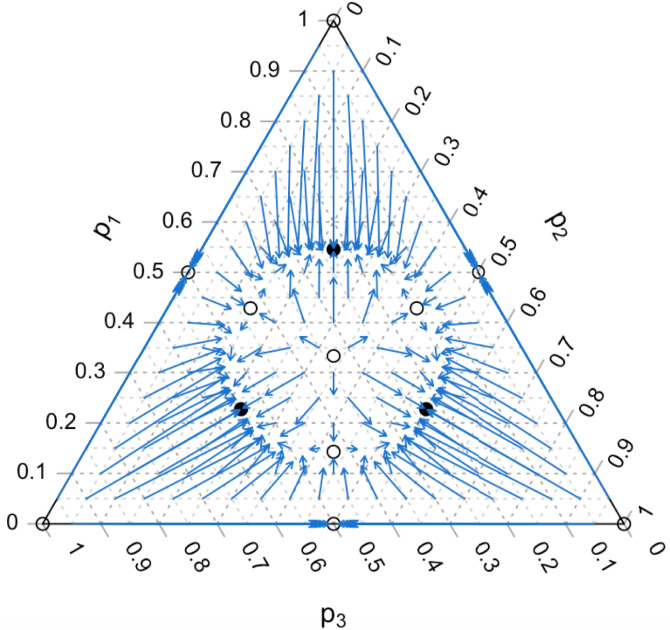
Dynamics with *n* = 5 role models and *m* = 3 variants, where D″′=−1.5, D″=0.9, D′=1.9, and D=−0.5. The base of each arrow is at the starting point $\left(p_{1}^{\left(0\right)},p_{2}^{\left(0\right)},p_{3}^{\left(0\right)}\right)$, and the top of the arrow is at the ending point after 20 generations. Stable equilibria are shown as filled circles, and unstable equilibria are shown as open circles.

With *n* = 5 role models and *m* = 3 variants, there are four distinct, nonzero conformity coefficients (SI Appendices B and E - Table S2). In this case we can find six interior equilibria at which two variant frequencies are equal, and our numerical analysis indicates that unlike the case of *n* = 4 and *n* = 3 role models with *m* = 3 variants, these equilibria may be stable (Fig. 2). We cannot exclude the existence of other equilibria where all of $p_{1},p_{2}$, and *p*_3_ are nonzero and unequal.

## Generalizations to *n* Role Models and *m* Variants

### Equilibria and Local Stability.

In all the above examples, there are corner equilibria such as (1,0,…,0), boundary equilibria such as $\left(\frac{1}{2},\frac{1}{2},0,\ldots ,0\right)$, etc., up to a central symmetric polymorphism $\left(\frac{1}{m},\frac{1}{m},\ldots ,\frac{1}{m}\right)$. This is the case in general with *n* role models and *m* variants. The proof is in *SI Appendix*, section C.

In *SI Appendix*, section D, we show that the polymorphic equilibrium $p^{*}=(\frac{1}{m},\frac{1}{m},\ldots ,\frac{1}{m})$ is locally stable iff

[15] $$|1+\frac{m}{m-1}\alpha |<1,$$

where

[16] $$\alpha =\frac{1}{n\cdot m^{n-1}}\sum_{x}D_{i}(x)\frac{n!}{x_{1}!x_{2}!\cdots x_{m}!}\cdot x_{i}\;\;i=1,\ldots ,m,$$

and that all corners are locally stable iff $D_{1}(x)>0$ for x=(n−1,1,0,0,…,0).

### Rationale for Multiple Conformity Coefficients: Classifying *D*(*x*).

With *n* role models and *m* variants, there may be many different conformity coefficients D(x). We propose the following rationale to reduce this number by using a single coefficient for each sample configuration, ***x***. Let *r* be the number of variants present in at least one role model in the sample ***x***. Then the average representation of a variant that is present in the sample is $\frac{n}{r}$ (which may or may not be an integer). For i=1,2,…,m, we can then write

[17a] $$P(A_{i}|x)=\frac{x_{i}}{n}+\frac{D_{i}(x)}{n}=\frac{x_{i}}{n}+\frac{g_{i}(x)d(x)}{n},$$

where

[17b] $$\text{​​​​​}g_{i}(x)\text{​}=\text{​}\{\begin{array}{ll} 0 & \text{​​​if}\;x_{i}\in \text{I}\;\text{I}=\{x:x=0,\frac{n}{r},n\}\\ \frac{x_{i}}{\sum_{z\in \text{II}}z} & \text{​​​if}\;x_{i}\in \text{II}\;\text{II}=\{x:\frac{n}{r}<x<n\}\\ -\frac{x_{i}^{-1}}{\sum_{z\in \text{III}}z^{-1}} & \text{​​​if}\;x_{i}\in \text{III}\;\text{III}=\{x:0\text{​}<\text{​}x<\frac{n}{r}\}. \end{array}$$

Here d(x) is the constant conformity coefficient that depends on the sample configuration ***x*** (and d(x) is equivalent to d(y) if ***y*** has all the same values $x_{1},x_{2},\ldots ,x_{m}$ but in another order).

The intuition behind Eq. **17** can be illustrated with an example. Suppose that a sample of *n* = 100 role models contains 25 *A*_1_, 20 *A*_2_, 20 *A*_3_, 10 *A*_4_, 10 *A*_5_, 10 *A*_6_, and 5 *A*_7_ so that x=(25,20,20,10,10,10,5,0,…,0). Then the probabilities of adoption, according to Eq. **17**, are $A_{1}:\frac{25}{100}+\left(\frac{25}{65}\right)\frac{d(x)}{100},\;A_{2}:\frac{20}{100}+\left(\frac{20}{65}\right)\frac{d(x)}{100},\;A_{3}:\frac{20}{100}+\left(\frac{20}{65}\right)\frac{d(x)}{100}$, $A_{4}:\frac{10}{100}-0.2\frac{d(x)}{100},\;A_{5}:\frac{10}{100}-0.2\frac{d(x)}{100},\;A_{6}:\frac{10}{100}-0.2\frac{d(x)}{100}$, and $A_{7}:\frac{5}{100}-0.4\frac{d(x)}{100}$.

Here, the average representation of *A_i_* in the role models is (100 role models)/(7 variants) ≈14 individuals. The positive conformity coefficient ($+\frac{d(x)}{100}$) is partitioned among the three variants that are represented in more than 14 role models, allocated according to their frequencies in this subsample of 65 (25 + 20 + 20). The negative conformity coefficient ($-\frac{d(x)}{100}$) is partitioned among the four variants that occur in fewer than 14 individuals. The resulting conformity coefficients are negatively correlated with occurrence in the sample, so since 5 is half of 10, variant *A*_7_ has double the negative conformity coefficient of *A*_4_, *A*_5_, and *A*_6_.

A different sample of *n* = 100 role models might include 99 *A*_1_ and 1 *A*_2_. In this case, ***x*** differs from the previous example and is given by (99,1,0,…,0), so the probabilities of adoption are $A_{1}:\frac{99}{100}+\frac{d(x)}{100}$ and $A_{2}:\frac{1}{100}-\frac{d(x)}{100}$. It is important to note that d(x) in the current example need not equal d(x) of the previous example. We incorporate this flexibility for two reasons. First, it seems unrealistic to assume that the extent to which an individual conforms to variant *A*_1_ when it is present in 99 individuals would be the same as when it is present in 25 individuals; e.g., *A*_1_ might be perceived as less attractive as it becomes too frequent in the population. Second, the upper bound of d(x) in the example with 99 *A*_1_ is 1, but this upper bound does not seem realistic for d(x) in the first example, with 25 *A*_1_. If d(x) were at most 1, then variant *A*_1_ in the first example could be adopted with a probability of at most ~0.2538 (differing only slightly from random copying, where *A*_1_ would be adopted with probability 0.25). In reality, a conformist might adopt *A*_1_ in the first example with a much greater probability. The bounds on d(x) are shown below.

For a given sample $x=(x_{1},x_{2},\ldots ,x_{m})$, the probability $P(A_{i}|x)$ must be between 0 and 1. Therefore,

[18] $$-\sum_{z\in \text{II}}z<d(x)<\sum_{z\in \text{II}}z(\frac{n}{\underset{x_{i}\in x}{\max}x_{i}}-1),$$

which is proved in *SI Appendix*, section F.

Ultimately, the recursion in *p_i_* with the system given by Eq. **17** is

[19] $$\begin{array}{ll} p_{i}\prime & =p_{i}+\frac{1}{n}\sum_{x}d(x)g_{i}(x)\frac{n!}{x_{1}!x_{2}!\cdots x_{m}!}p_{1}^{x_{1}}p_{2}^{x_{2}}\cdots p_{m}^{x_{m}}. \end{array}$$

In *SI Appendix*, section E, Eq. **17** is applied to the cases of *m* = 3 variants and *n* = 4 or 5 role models, and the previous choices of D(x) in these cases are shown to be consistent with the formulation in Eq. **17**. However, the system given by Eq. **17** is not the only parsimonious formulation of conformity coefficients, and another possible classification of D(x) is offered in *SI Appendix*, section G.

### Global Stability of Equilibria.

In *SI Appendix*, section H, we prove the following result concerning global convergence of Eq. **19** to an equilibrium.

Result 5. With n role models and m variants, there is global convergence to an equilibrium provided d(x)>0 for all x (i.e., there is entirely conformist transmission). If there are initially l≥1 variants all at the maximum variant frequency, $\max_{1\leq i\leq m}p_{i}$, then at equilibrium, the frequencies of these l variants are $\frac{1}{l}$, and all others are zero.

**Fig. 3.**
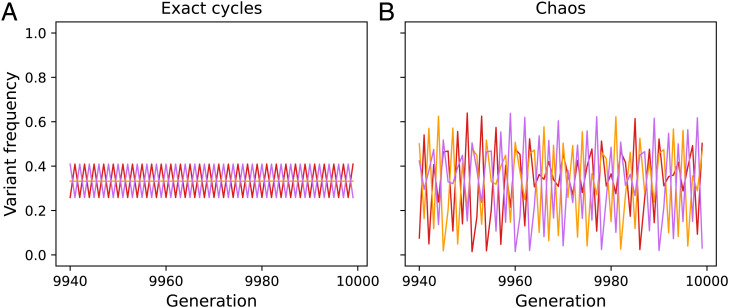
Variant frequencies over time for *n* = 15 role models and *m* = 3 variants. In both *A* and *B*, Eq. **19** is iterated from initial variant frequencies $p_{1}^{\left(0\right)}=0.48$ (red), $p_{2}^{\left(0\right)}=0.501$ (orange), and $p_{3}^{\left(0\right)}=0.019$ (purple). (*A*) d(x)=−5.9 for all x and (*B*) $d(x)=-\sum_{z\in \text{II}}z+0.1$ (where $\text{II}=\{x:\frac{n}{r}<x<n\}$; Eq. **17b**). Both simulations ran for 10,000 generations, and the last 60 are plotted. In *A*, there is an exact two-generation cycle between p≈(0.259,0.332,0.409) and p≈(0.409,0.332,0.259). In *B*, there are chaotic fluctuations around an average frequency (calculated over the last 5,000 generations) of p≈(0.334,0.332,0.334).

On the other hand, if d(x)<0 for some ***x*** (i.e., there is anticonformist transmission), there need not be global convergence to an equilibrium. Instead, stable cycles or chaos can occur. Examples of such cycles and chaos are shown in Fig. 3.

In ref. 25, with *m* = 2 variants and *n* = 5 role models, a stable cycle could occur around an average frequency vector $p^{*}=(0.5,0.5)$ (figure 2c in ref. 25). In Fig. 3*A*, there are *m* = 3 variants and *n* = 15 role models, and the stable cycle is around an average frequency vector $p^{*}\approx (0.334,0.332,0.334)$. However, if *m* = 3 and *n* is reduced to 5 role models, cycles do not seem to occur (Table 2, row 5). Thus, while cycles are not unique to the polychotomous model, they can take different forms and occur under different conditions from the dichotomous model.

## Discussion

Our model of conformity to a polychotomous trait generalizes the dichotomous trait model of Boyd and Richerson (2), which has been widely used in previous theoretical studies of conformity (14–21, 24, 26). Dichotomous traits have two variants, such as pro/con, skilled/unskilled, or cooperate/defect, whereas polychotomous traits, such as baby names, art motifs, or birdsong syllables, have three or more variants.

It has been suggested that dichotomous trait conformity models provide a reasonable approximation to polychotomous trait models, particularly when a novel variant enters the population (15). The variant of interest can be considered type *A* and all other variants grouped into type *B*, and under conformity it seems intuitive that *A* will decrease in frequency, while under anticonformity, *A* will increase. However, we find that the long-term behavior predicted by dichotomous trait models and polychotomous trait models can differ greatly, particularly under anticonformist transmission (Table 2). For example, with strong anticonformity and *n* = 5 role models sampled from the adult generation, stable cycles can occur in the dichotomous trait model (25), while convergence to an equilibrium appears to occur in the polychotomous trait model (Table 2, row 5). Under sufficiently weak anticonformity, the dichotomous trait model predicts convergence to an equilibrium of $\frac{1}{2}A$ and $\frac{1}{2}B$ (25), whereas the equilibrium frequency of *A* in the polychotomous trait model can be much smaller—for example, if the number of variants in the population is 1,000, the frequency of *A* can be 0.001 at equilibrium (e.g., *Result 3iii*). Anticonformist transmission has been observed empirically (3, 31), and some have suggested that humans are generally more likely to exhibit anticonformist than conformist bias (31). Thus, it is important to be able to accurately model population dynamics under anticonformity with two or more variants.

Under conformity, we show here that the dichotomous trait model may or may not provide a reasonable approximation to the polychotomous trait model depending on the question that is asked. If the question is “What happens to a new variant, *A*, over time?” then both the dichotomous and polychotomous models produce the same result: *A* will be lost. However, if the question is “What are the frequencies of variants at equilibrium?” then for the dichotomous trait model, the simple answer is “*A* will not be present,” while the polychotomous trait model provides a more informative answer: “If initially the frequencies of l≥1 variants equal the maximum variant frequency in the population, then these l variants reach frequencies of $\frac{1}{l}$ at equilibrium.”

A small number of studies have modeled conformity to a polychotomous trait, e.g., refs. 18, 23, 27. In these, conformity is incorporated using a single coefficient, and the formula for conformist transmission does not include the number of sampled role models, *n*. In our model with *n* = 3 role models, there is also a single conformity coefficient, *D*, and the dynamics are relatively straightforward. Under conformity, the variant(s) with the highest frequency will increase until it (they) cannot increase further (*Result 5*), while under anticonformity, there is global convergence to an equilibrium in which all *m* variants in the population are present at equal frequencies, namely, $p^{*}=(\frac{1}{m},\ldots ,\frac{1}{m})$. Anticonformity entails that more common variants are adopted at a rate less than their frequency; thus, any population state where not all variant frequencies equal $\frac{1}{m}$ will result in the more common variant frequencies (above $\frac{1}{m}$) decreasing and the less common frequencies (below $\frac{1}{m}$) increasing.

In our model with *n* > 3 role models, however, the dynamics may not be the same as with *n* = 3. Previous empirical research has shown that individuals’ levels of conformity to a dichotomous trait can change as the number of observed individuals changes (9), suggesting that having different formulas for different *n* values might be useful. The same finding was not observed for a polychotomous trait in ref. 9, although this might simply be due to the small numbers of role models that were used. In addition, the relationship between the numbers of role models and Aschian conformity, defined as “the overriding of personal knowledge or behavioral dispositions by countervailing options observed in others” (ref. 32, p. 34), has been widely investigated. For example, Bond (33) conducted a metaanalysis of 125 experiments of Aschian conformity to a polychotomous trait with *m* = 3 variants and found that the relationship between conformity and the number of observed individuals was more complex than previously assumed. Overall, therefore, allowing levels of conformity and anticonformity to vary with *n* for both a polychotomous and a dichotomous trait may add realism to theoretical models.

We find that if individuals are purely conformist, the long-term population dynamics are the same for any number of role models, *n* (i.e., there is global convergence to an equilibrium in which the l≥1 variants that were all initially present at the maximum frequency stabilize at a frequency of $\frac{1}{l}$ and all other variants disappear). With anticonformity, however, the population dynamics can change greatly as *n* changes. Anticonformity amplifies the frequencies of rare variants, which, in the case of *n* = 3 role models, for example, causes global convergence to the central polymorphic equilibrium $p^{*}=(\frac{1}{m},\ldots ,\frac{1}{m})$. However, as *n* increases, anticonformity can become stronger; for example, if d(x)=D for all ***x*** in Eq. **17**, the lower bound of *D* is –2 with *n* = 3 but –6 with *n* = 15 (Fig. 3*A*). With strong enough anticonformity, a rare variant may be favored so strongly that its frequency overshoots the point $\left(\frac{1}{m},\ldots ,\frac{1}{m}\right)$, and subsequently, this common variant can be so strongly disfavored that it returns to its previous frequency. This process can continue indefinitely, producing a stable cycle (Fig. 3*A*). In addition, Fig. 3*B* shows that if *n* = 15 and d(x) is near its lower bound for each ***x*** (thus, not all d(x) are equal), anticonformity can be so strong that the dynamics exhibit chaos rather than stable cycling. Similarly, in the dichotomous trait model we showed that with large enough *n* and strong enough anticonformity, cycles or chaos could occur (25). Thus, the number of role models sampled plays an important role in the evolutionary dynamics.

Another difference between our model and previous models of conformity to a polychotomous trait is that the conformity coefficients are functions of ***x***, the configuration of sampled role models (i.e., how many role models have each variant). For example, in Eq. **17a** with *m* = 3 variants, the probability of adopting a variant in, say, 99% of role models is at most 100% (an increase of 1%), so if there were only one conformity coefficient for all x, the probability of adopting a variant present in 70% of role models would be at most 71% (an increase of 1%). Instead, allowing a conformist to adopt a variant at 70% frequency with a probability that is greater than 71% seems more realistic.

Moreover, there is some empirical evidence that individuals’ levels of conformity can change depending on the configuration of sampled role models for a dichotomous trait, so the same may be true for a polychotomous trait. In ref. 8, female fruit flies observed different numbers of other females copulating with males painted pink or green. When they observed 60% of role models copulating with one type of male, they copulated with that type of male at a frequency greater than 60% (conformity), but when they observed 83% of role models copulating with one type of male, they displayed anticonformity (by our definition, following ref. 2). It seems reasonable to expect that a similar trend could occur for a polychotomous trait; for example, an up-and-coming fashion observed in a slight majority of individuals might be adopted with high frequency, but once the same fashion is adopted by a large majority of people it might become less appealing and be adopted with a lower frequency.

Therefore, a key feature of the present model is that with n≥4 role models, conformity coefficients can vary in sign and magnitude depending on the role model state, ***x***. Even in the simplest case of a polychotomous trait with n≥4 role models, namely, *n* = 4 and *m* = 3 variants, dynamics become much more complex than in the case of *n* = 3 role models. For example, the population may converge to a boundary equilibrium such as $p^{*}=(\frac{1}{2},\frac{1}{2},0)$ even if all variants’ initial frequencies are nonzero and unequal (Fig. 1), which was not possible in the case with *n* = 3 role models. Moreover, there can be three new, asymmetric interior equilibria given by Eq. **14**. A necessary but not sufficient condition for existence of these equilibria is that anticonformity occurs for some role model states and conformity occurs for others. If transmission were entirely conformist or entirely anticonformist, there would be no opposing forces that would lead to the existence of an asymmetric equilibrium (as this model does not include selection). Similarly, in ref. 25, it was shown that in Boyd and Richerson’s dichotomous trait conformity model, asymmetric polymorphic equilibria could exist without selection provided conformity and anticonformity occurred for different role model states (shown in figure 1 in ref. 25).

The range of complex dynamics that characterize polychotomous trait systems with n=5,6,… role models and conformity coefficients that vary in sign remains to be explored. For example, whereas three asymmetric interior equilibria could exist with *n* = 4 role models and *m* = 3 variants, with *n* = 5 and *m* = 3, there could be six (Fig. 2), although there may be more. In addition, it would be interesting to investigate the effects of selection on the dynamics of these models. Incorporating selection into dichotomous trait models of conformity produced novel asymmetric equilibria and eliminated the symmetric equilibrium $p^{*}=(\frac{1}{2},\frac{1}{2})$, so if selection were included in polychotomous trait models, the symmetric equilibrium $p^{*}=(\frac{1}{m},\frac{1}{m},\ldots ,\frac{1}{m}$) may no longer exist.

It is often claimed that conformity homogenizes groups (i.e., reduces within-group variation) and increases between-group differences, which facilitates group selection (2, 14, 34–37). In our model, if there is purely conformist transmission and initially one variant is most common, then there is global convergence to fixation of this variant, and the population becomes homogeneous. However, if more than one variant is initially present at the highest frequency, then the population is not homogeneous at equilibrium. Our model included only one population or group, and the effect of conformity on between-group differences requires further exploration. In the dichotomous trait conformity model, in some cases, introducing or increasing conformity could decrease between-group differences (25), and whether the same is true in the polychotomous trait model remains to be investigated.

Furthermore, many studies of conformity, including the present, have omitted individual-level variation in conformity, and future research could explore the dynamics when some members of the population have greater tendencies to conform or anticonform than others. Moreover, incorporating nonrandom choices of the *n* role models (e.g., family, close friends, or prestigious individuals) would likely produce different population dynamics that would be interesting to explore. Finally, temporal variation in conformity coefficients has recently been incorporated into the dichotomous trait model (26), and it would be interesting to explore the consequences of such variation in the polychotomous case.

## Supplementary Material

Supplementary File

## Data Availability

The code for all simulations is now publicly available at https://github.com/kaleda/polychotomous-conformity (38; 39).
