# Supplementary material for: Polychotomous traits and evolution under conformity
Source: Proc Natl Acad Sci U S A. 2022 Sep 19;119(39):e2205914119. doi: 10.1073/pnas.2205914119 (PMC9522326; doi:10.1073/pnas.2205914119)
Supplement: Supplementary File [file pnas.2205914119.sapp.pdf]

PNAS
www.pnas.org

Supplementary Information for

Polychotomous traits and evolution under conformity

Kaleda K. Denton, Uri Liberman, and Marcus W. Feldman

Corresponding Author: Marcus W. Feldman.
E-mail: mfeldman@stanford.edu

This PDF file includes:

Supplementary text
Tables S1 to S2
SI References

Supporting Information Text

Supplementary Information A: Proofs of Results 1, 2, 3, and 4

Proof of Result 1, $n = 3, m = 3$. To determine equilibria of the recursion system (11) when $D \neq 0$, let $p_i' = p_i$, so

$$0 = p_i[p_i - (p_1^2 + p_2^2 + p_3^2)] \qquad i = 1, 2, 3. \tag{A1}$$

If $p_1 = 0$, Eq. (A1) with $i = 1$ is satisfied, and $p_3 = 1 - p_2$, in which case Eq. (A1) with $i = 2$ becomes

$$0 = p_2(1 - p_2)(2p_2 - 1). \tag{A2}$$

Solutions to (A2) are $p_2 = 0, 1,$ and $\frac{1}{2}$, so the equilibria are $\boldsymbol{p}^* = (0, 0, 1)$, $(0, 1, 0)$, and $(0, \frac{1}{2}, \frac{1}{2})$. Repeating this process but initially assuming that $p_2 = 0$ or $p_3 = 0$ rather than $p_1 = 0$ gives the additional equilibria $\boldsymbol{p}^* = (1, 0, 0)$, $(\frac{1}{2}, 0, \frac{1}{2})$, and $(\frac{1}{2}, \frac{1}{2}, 0)$. Finally, if $p_i \neq 0$ for all i, then by Eq. (A1), we have

$$p_1^2 + p_2^2 + p_3^2 = p_i \qquad i = 1, 2, 3. \tag{A3}$$

Hence, p_1, p_2, and p_3 all equal the same quantity on the left-hand side, and since $p_1 + p_2 + p_3 = 1$, we have $p_1 = p_2 = p_3 = \frac{1}{3}$.

To explore local stability of these equilibria, we distinguish between internal and external local stability. Internal stability is evaluated by perturbing an equilibrium within its boundary (where at least one variant's frequency remains at zero), in which case the analysis is equivalent to the dichotomous-trait analysis in (1), where with $n = 3$ role models, it was shown that corners are globally stable if $D > 0$ and boundary equilibria are globally stable if $D < 0$.

External stability of a corner or a boundary equilibrium is tested by perturbing the equilibrium towards the interior, so that $p_1, p_2, p_3 > 0$. For the equilibrium $\boldsymbol{p}^* = (0, 0, 1)$, set $p_1 = \varepsilon_1$, $p_2 = \varepsilon_2$, and $p_3 = 1 - \varepsilon_1 - \varepsilon_2$, where $\varepsilon_1, \varepsilon_2$ are small enough that $o(\varepsilon_i)$ terms can be neglected. Then, from Eq. (11),

$$\varepsilon_i' = \varepsilon_i(1 - D) \qquad i = 1, 2, \tag{A4}$$

where $-2 < D < 1$ (see below Eqs. (9)). Thus, if $D > 0$, then corner equilibria are locally stable (i.e., $\varepsilon_i' < \varepsilon_i$ for $i = 1, 2$). For external local stability of the boundary equilibrium $\boldsymbol{p}^* = (0, \frac{1}{2}, \frac{1}{2})$, set $p_1 = \varepsilon_1$, $p_2 = \frac{1}{2} + \varepsilon_2$, and $p_3 = \frac{1}{2} - \varepsilon_1 - \varepsilon_2$. Then by Eq. (11),

$$\varepsilon_1' = \varepsilon_1(1 - \tfrac{1}{2}D), \tag{A5}$$

so $D > 0$ is required for external stability. However, $D > 0$ entails that boundary equilibria are internally unstable. A similar analysis with $p_1 = \frac{1}{3} + \varepsilon_1$, $p_2 = \frac{1}{3} + \varepsilon_2$, and $p_3 = \frac{1}{3} - \varepsilon_1 - \varepsilon_2$, gives

$$\varepsilon_i' = \varepsilon_i(1 + \tfrac{1}{3}D) \qquad i = 1, 2 \tag{A6}$$

so the interior equilibrium $(\frac{1}{3}, \frac{1}{3}, \frac{1}{3})$ is locally stable if D is negative; that is, $-2 < D < 0$.

To sum up, as in the case with $m = 2$ variants, only one parameter D is needed to determine all conformity vectors $\boldsymbol{D}(\boldsymbol{x})$ and, depending on whether $D > 0$ or $D < 0$, either the "corners" or the central polymorphism, respectively, are locally stable.

Proof of Result 2, $n = 3, m \geq 3$ local stability. Here we analyze the local stability of the equilibria of the recursion (12), deriving Result 2. Without loss of generality, take i to be 1, so Eq. (12) becomes

$$p_1' = p_1 + Dp_1 \left[p_1 - \sum_{j=1}^{m} p_j^2 \right]. \tag{A7}$$

For the equilibrium $\boldsymbol{p}^* = (1, 0, \ldots, 0)$, set $p_1 = 1 - \varepsilon_1$ and $p_i = \varepsilon_i$ for $i = 2, \ldots, m$, where ε_i are small, $\varepsilon_i \geq 0$, and $\sum_{i=2}^{m} \varepsilon_i = \varepsilon_1 > 0$. Near $\boldsymbol{p}^* = (1, 0, \ldots, 0)$, the linear approximation to Eq. (A7) gives

$$\varepsilon_1' = \varepsilon_1(1 - D). \tag{A8}$$

Therefore, as $-2 < D < 1$, corners are locally stable if $D > 0$.

For the interior equilibrium $\boldsymbol{p}^* = (\frac{1}{m}, \frac{1}{m}, \ldots, \frac{1}{m})$, set $p_i = \frac{1}{m} + \varepsilon_i$ for $i = 1, 2, \ldots, m$. Here, ε_i are small, $\sum_{i=1}^{m} \varepsilon_i = 0$, and assume $\varepsilon_1 > 0$. Then the linear approximation to Eq. (A7) gives

$$\varepsilon_1' = \varepsilon_1 \left(1 + \frac{1}{m} D \right). \tag{A9}$$

Thus, $\boldsymbol{p}^* = (\frac{1}{m}, \frac{1}{m}, \ldots, \frac{1}{m})$ is locally stable if $-2m < D < 0$. However, as $-2 < D < 1$ and $m \geq 3$, $-2m < D$. Thus, the condition for local stability is $D < 0$.

Finally, we must show that all other equilibria are unstable. Perturb a boundary equilibrium $\boldsymbol{p}^* = (\frac{1}{\ell}, \ldots, \frac{1}{\ell}, 0, \ldots, 0)$ within its boundary, so that

$$p_1 = \frac{1}{\ell} + \varepsilon_1, \ldots, p_\ell = \frac{1}{\ell} + \varepsilon_\ell, \; p_{\ell+1} = 0, \ldots, p_m = 0, \tag{A10}$$

where for $i = 1, \ldots, \ell$, the frequencies ε_i are small, $\sum_{i=1}^{\ell} \varepsilon_i = 0$, and assume $\varepsilon_1 \neq 0$. Then the linear approximation to Eq. (A7) gives

$$\varepsilon_1' = \varepsilon_1 \left(1 + \frac{1}{\ell} D\right). \tag{A11}$$

Thus, as $-2 < D < 1$ and $\ell \geq 2$, boundary equilibria such as $\boldsymbol{p}^* = (\frac{1}{\ell}, \ldots, \frac{1}{\ell}, 0, \ldots, 0)$ are stable within their boundary if $D < 0$.

Now, perturb a boundary equilibrium, for example $\boldsymbol{p}^* = (0, \ldots, 0, \frac{1}{\ell}, \ldots, \frac{1}{\ell})$ into the interior. Set

$$p_1 = \varepsilon_1, \ldots, p_{m-\ell} = \varepsilon_{m-\ell},\ p_{m-\ell+1} = \frac{1}{\ell} + \varepsilon_{m-\ell+1}, \ldots, p_m = \frac{1}{\ell} + \varepsilon_m. \tag{A12}$$

Assume that for $i = 1, \ldots, m-\ell$, the frequencies ε_i are small, $\varepsilon_i \geq 0$, and $\sum_{i=1}^{m-\ell} \varepsilon_i > 0$ so that the perturbation is into the interior. Then, $\sum_{i=m-\ell+1}^{m} \varepsilon_i = -\sum_{i=1}^{m-\ell} \varepsilon_i$ is the net frequency change away from the boundary. If $\varepsilon_1 > 0$ then the linear approximation to Eq. (A7) gives

$$\varepsilon_1' = \varepsilon_1 \left(1 - \frac{1}{\ell} D\right). \tag{A13}$$

Thus, as $-2 < D$ and $\ell \geq 2$, stability of boundary equilibria requires $D > 0$, but if $D > 0$, then the boundary equilibria are unstable within their boundaries by (A11). Overall, therefore, boundary equilibria are not locally stable.

Proof of Result 3, $n = 3, m \geq 3$ global stability. Without loss of generality, assume initially that $p_1 = \max_{1 \leq i \leq m} p_i < 1$ and $p_2 < p_1$ (although p_i for $i = 3, \ldots, m$ may equal p_1). Let $D < 0$. The equilibrium $\boldsymbol{p}^* = (\frac{1}{m}, \frac{1}{m}, \ldots, \frac{1}{m})$ is globally stable iff

$$\text{(a) } p_1' < p_1 \qquad \text{(b) } p_1' > p_2' \qquad \text{(c) } p_1' - p_2' < p_1 - p_2.$$

By Eq. (12),

$$\frac{p_1'}{p_1} = 1 + D \sum_{j \neq 1} p_j (p_1 - p_j). \tag{A14}$$

As $p_1 > p_2$ and $p_1 \geq p_j$ for $j = 3, \ldots, m$, $\sum_{j \neq 1} p_j (p_1 - p_j) > 0$. Therefore, if $-2 < D < 0$ (see below Eqs. (9)) then $\frac{p_1'}{p_1} < 1$ and (a) holds. Subtracting Eq. (12) with $i = 2$ from Eq. (12) with $i = 1$ gives

$$p_1' - p_2' = (p_1 - p_2) \left\{ 1 + D \left[p_1(1 - p_1) + p_2(1 - p_2) - \sum_{j \neq 1,2} p_j^2 \right] \right\}. \tag{A15}$$

Since $p_1(1 - p_1) + p_2(1 - p_2) < \frac{1}{2}$ (because $p_i(1 - p_i) \leq \frac{1}{4}$ and $p_2 < p_1$) and $\sum_{j \neq 1,2} p_j^2 \geq 0$, we have $p_1(1 - p_1) + p_2(1 - p_2) - \sum_{j \neq 1,2} p_j^2 < \frac{1}{2}$. Thus, as the lower bound of D is -2, we have that $p_1' - p_2' > 0$ and hence (b) holds. Rearranging Eq. (A15),

$$p_1' - p_2' = (p_1 - p_2) \left\{ 1 + D \left[2p_1 p_2 + \sum_{j \neq 1,2} p_j (p_1 + p_2 - p_j) \right] \right\}. \tag{A16}$$

Since $p_1 - p_j \geq 0$ for $j = 3, \ldots, m$, we have $2p_1 p_2 + \sum_{j \neq 1,2} p_j (p_1 + p_2 - p_j) > 0$. Therefore, if $D < 0$, then $(p_1' - p_2') < (p_1 - p_2)$ and (c) holds.

Denote the population state at generation t by $\boldsymbol{p}^{(t)} = (p_1^{(t)}, p_2^{(t)}, \ldots, p_m^{(t)})$ for $t = 0, 1, 2, \ldots$, and recall that we have assumed, without loss of generality, that $p_1^{(0)} = \max_{1 \leq i \leq m} p_i^{(0)} < 1$, that $p_2^{(0)} < p_1^{(0)}$, and that $p_i^{(0)} \leq p_1^{(0)}$ for $i = 3, \ldots, m$. Then, applying (a), (b), and (c), we can conclude that $p_1^{(t)} = \max_{1 \leq i \leq m} p_i^{(t)} < 1$ for $t = 0, 1, 2, \ldots$ and that both $\{p_1^{(t)}\}_{t=0}^{\infty}$ and $\{p_1^{(t)} - p_2^{(t)}\}_{t=0}^{\infty}$ are monotone decreasing and bounded. By symmetry, if $p_i^{(0)} < p_1^{(0)}$ for any $i = 3, \ldots, m$, then $\{p_1^{(t)} - p_i^{(t)}\}_{t=0}^{\infty}$ is also monotone decreasing and bounded, whereas if $p_i^{(0)} = p_1^{(0)}$ for any $i = 3, \ldots, m$, then $p_1^{(t)} - p_i^{(t)} = 0$ for all $t = 0, 1, \ldots$. Hence, $\boldsymbol{p}^{(t)} = (p_1^{(t)}, p_2^{(t)}, \ldots, p_m^{(t)}) \xrightarrow[t \to \infty]{} \boldsymbol{p}^{(\infty)}$ where $\boldsymbol{p}^{(\infty)}$ is an equilibrium. Specifically, $\boldsymbol{p}^{(\infty)} = (\frac{1}{m}, \ldots, \frac{1}{m})$ because by Result 2, if $D < 0$, then $\boldsymbol{p}^* = (\frac{1}{m}, \ldots, \frac{1}{m})$ is the only locally stable equilibrium while all others are unstable.

A similar argument can be applied to show Result 3 parts *(i)* and *(ii)* if $0 < D < 1$.

Proof of Result 4, $n = 4, m = 3$ local stability. First, we will find interior equilibria with $p_1, p_2, p_3 > 0$ other than $\boldsymbol{p}^* = (\frac{1}{3}, \frac{1}{3}, \frac{1}{3})$. Subtracting Eq. (13) with $i = 2$ from Eq. (13) with $i = 1$, at equilibrium

$$0 = (p_1 - p_2)\{D'[(1 - p_1)(2p_1 - 1) + (1 - p_2)(2p_2 - 1) + 1 - 2p_1p_2 - 3p_1p_2p_3] + \tfrac{9}{2}p_1p_2p_3D\}. \quad \text{[A17]}$$

Hence either $p_1 = p_2$, or $p_1 \neq p_2$ and

$$0 = D'[(1 - p_1)(2p_1 - 1) + (1 - p_2)(2p_2 - 1) + 1 - 2p_1p_2 - 3p_1p_2p_3] + \tfrac{9}{2}p_1p_2p_3D. \quad \text{[A18]}$$

Now subtracting Eq. (13) with $i = 3$ from Eq. (13) with $i = 1$ and assuming $p_1 \neq p_3$ gives

$$0 = D'[(1 - p_1)(2p_1 - 1) + (1 - p_3)(2p_3 - 1) + 1 - 2p_1p_3 - 3p_1p_2p_3] + \tfrac{9}{2}p_1p_2p_3D. \quad \text{[A19]}$$

Subtracting Eq. (A19) from Eq. (A18) gives $0 = D'(p_2 - p_3)$. If $D' \neq 0$, then $p_2 = p_3$, in which case $p_1 = 1 - 2p_2$. Then, by Eq. (A18), if $p_1, p_2, p_3 > 0$ and $p_2 \neq \frac{1}{2}$, we find $p_2 = \frac{2D'}{2D' - 3D}$. Therefore, by symmetry, there are three possible equilibria:

$$\begin{aligned}(p_1^*, p_2^*, p_3^*) = &\left(\frac{-2D' - 3D}{2D' - 3D}, \frac{2D'}{2D' - 3D}, \frac{2D'}{2D' - 3D}\right),\\ &\left(\frac{2D'}{2D' - 3D}, \frac{-2D' - 3D}{2D' - 3D}, \frac{2D'}{2D' - 3D}\right),\\ &\left(\frac{2D'}{2D' - 3D}, \frac{2D'}{2D' - 3D}, \frac{-2D' - 3D}{2D' - 3D}\right),\end{aligned} \quad \text{[A20]}$$

which are shown in Eq. (14) of the main text.

Second, we will evaluate local stability of the equilibria with $n = 4$ role models and $m = 3$ variants. The proof that the corners are locally stable if $D' > 0$ follows that of Result 1. The condition for internal local stability of the boundary equilibria $(\frac{1}{2}, \frac{1}{2}, 0), (\frac{1}{2}, 0, \frac{1}{2}), (0, \frac{1}{2}, \frac{1}{2})$ is $D' < 0$, as shown in (1) with $m = 2$ variants. To assess external local stability of these equilibria, take $D' < 0$, $p_1 = \frac{1}{2} + \varepsilon_1$, $p_2 = \frac{1}{2} + \varepsilon_2$ and $p_3 = -\varepsilon_1 - \varepsilon_2$, and neglect terms $O(\varepsilon_i^2)$. From Eq. (13), in the next generation,

$$\begin{bmatrix}\varepsilon_1'\\ \varepsilon_2'\end{bmatrix} = \begin{bmatrix}1 + \frac{1}{16}(2D' - 3D) & -\frac{3}{16}(2D' + D)\\ -\frac{3}{16}(2D' + D) & 1 + \frac{1}{16}(2D' - 3D)\end{bmatrix}\begin{bmatrix}\varepsilon_1\\ \varepsilon_2\end{bmatrix}. \quad \text{[A21]}$$

The eigenvalues of the Jacobian in (A21) are $\lambda_1 = 1 + \frac{1}{2}D'$ and $\lambda_2 = 1 - \frac{1}{4}D' - \frac{3}{8}D$. Then $\lambda_1 > 1$ if $D' > 0$ while $\lambda_2 > 1$ if $2D' + 3D < 0$. However, because $D' < 0$ is required for internal local stability of these equilibria in the boundaries, we assume $D' < 0$. Hence, the condition for external instability is $\lambda_2 > 1$; that is, $2D' + 3D < 0$, which also guarantees existence of the interior equilibria in Eq. (14).

Near $\boldsymbol{p}^* = (\frac{1}{3}, \frac{1}{3}, \frac{1}{3})$, let $p_1 = \frac{1}{3} + \varepsilon_1$, $p_2 = \frac{1}{3} + \varepsilon_2$, $p_3 = \frac{1}{3} - \varepsilon_1 - \varepsilon_2$. From Eq. (13) with $i = 1$,

$$\varepsilon_1' = \varepsilon_1(1 + \tfrac{2}{9}D' + \tfrac{1}{6}D). \quad \text{[A22]}$$

Thus, $(\frac{1}{3}, \frac{1}{3}, \frac{1}{3})$ is stable if $-1 < 1 + \frac{2}{9}D' + \frac{1}{6}D < 1$, i.e., if $-36 < 4D' + 3D < 0$. But $4D' + 3D > -36$ under the range assumptions on D' and D, so the condition for stability is $4D' + 3D < 0$.

Finally, consider $p_1^* = p_2^* = \frac{2D'}{2D' - 3D}$, and set $p_1 = p_1^* + \varepsilon_1$, $p_2 = p_2^* + \varepsilon_2$, and $p_3 = 1 - 2p_1^* - \varepsilon_1 - \varepsilon_2$. Then from Eq. (13),

$$\begin{bmatrix}\varepsilon_1'\\ \varepsilon_2'\end{bmatrix} = \begin{bmatrix}1 + D'p_1^*(3p_1^* - 1) & D'(3p_1^* - 1)(p_1^* - 1)\\ D'(3p_1^* - 1)(p_1^* - 1) & 1 + D'p_1^*(3p_1^* - 1)\end{bmatrix}\begin{bmatrix}\varepsilon_1\\ \varepsilon_2\end{bmatrix}. \quad \text{[A23]}$$

The eigenvalues of the Jacobian in (A23) are $\lambda_1 = 1 + D'(3p_1^* - 1)$ and $\lambda_2 = 1 + D'(3p_1^* - 1)(2p_1^* - 1)$. Since $p_1^* = p_2^* < \frac{1}{2}$, the term $2p_1^* - 1$ will always be negative. Therefore, $\lambda_2 < 1$ if $D'(3p_1^* - 1) > 0$, whereas $\lambda_1 < 1$ if $D'(3p_1^* - 1) < 0$. The equilibria (14), i.e., (A20) are therefore always unstable. An example of the dynamics in this case is illustrated in Figure 1 of the main text.

Supplementary Information B: $n = 5$ role models and $m = 3$ variants

With $n = 5$ role models and $m = 3$ variants there are four coefficients $d(\boldsymbol{x})$ (see Eq. (17a)) that may be non-zero, corresponding to the cases $\boldsymbol{x} = (4, 1, 0), (3, 1, 1), (3, 2, 0)$, and $(2, 2, 1)$. Denote these conformity coefficients by D''', D'', D', and D, respectively (see Table S2 in SI E). Using the symmetry property $\mathscr{P}$(ii) (for example, $(3, 2, 0)$ has the same conformity coefficient as $(2, 3, 0)$, namely D') and Eqs. (4) and (17), the recursions are,

$$\begin{aligned}p_1' = p_1 &+ D'''[p_1(1 - p_1)(2p_1 - 1)(p_1^2 - p_1 + 1) + p_1(4p_2^3p_3 + 6p_2^2p_3^2 + 4p_2p_3^3)]\\ &+ 2D''p_1(2p_1^2p_2p_3 - p_2^3p_3 - p_2p_3^3) + 2D'[p_1^3(p_2^2 + p_3^2) - p_1^2(p_2^3 + p_3^3)] + 3D[p_1^2(p_2^2p_3 + p_2p_3^2) - 2p_1p_2^2p_3^2]\end{aligned} \quad \text{[B1a]}$$

$$\begin{aligned}p_2' = p_2 &+ D'''[p_2(1 - p_2)(2p_2 - 1)(p_2^2 - p_2 + 1) + p_2(4p_1^3p_3 + 6p_1^2p_3^2 + 4p_1p_3^3)]\\ &+ 2D''p_2(2p_2^2p_1p_3 - p_1^3p_3 - p_1p_3^3) + 2D'[p_2^3(p_1^2 + p_3^2) - p_2^2(p_1^3 + p_3^3)] + 3D[p_2^2(p_1^2p_3 + p_1p_3^2) - 2p_2p_1^2p_3^2]\end{aligned} \quad \text{[B1b]}$$

$$\begin{aligned}p_3' = p_3 &+ D'''[p_3(1 - p_3)(2p_3 - 1)(p_3^2 - p_3 + 1) + p_3(4p_2^3p_1 + 6p_2^2p_1^2 + 4p_2p_1^3)]\\ &+ 2D''p_3(2p_3^2p_2p_1 - p_2^3p_1 - p_2p_1^3) + 2D'[p_3^3(p_2^2 + p_1^2) - p_3^2(p_2^3 + p_1^3)] + 3D[p_3^2(p_2^2p_1 + p_2p_1^2) - 2p_3p_2^2p_1^2].\end{aligned} \quad \text{[B1c]}$$

To obtain the equilibria (i.e., solutions of $\boldsymbol{p}' = \boldsymbol{p}$), first let $p_1^* = 0$ and thus $p_3^* = 1 - p_2^*$. Eq. (B1b) becomes

$$0 = p_2^*(1 - p_2^*)(2p_2^* - 1)[D''' - p_2^*(1 - p_2^*)(D''' - 2D')], \qquad \text{[B2]}$$

which is the same as Eq. (10) in (1). Clearly $p_2^* = 0, 1$, and $\frac{1}{2}$ are all equilibria. If $p_2^* \neq 0, 1, \frac{1}{2}$, then there may be another two equilibria $\hat{p}$ and $1 - \hat{p}$ that are solutions of

$$0 = D''' - \hat{p}(1 - \hat{p})(D''' - 2D'). \qquad \text{[B3]}$$

Valid solutions to (B3), i.e., $0 < \hat{p} < 1$, exist if either

(i) $D''' > 0$, $D' < 0$, and $D''' < -\frac{2}{3}D'$, or

(ii) $D''' < 0$, $D' > 0$, and $D''' > -\frac{2}{3}D'$.

Proof.
The derivative of the right-hand side of (B3) with respect to $\hat{p}$ is $(2\hat{p} - 1)(D''' - 2D')$.
Case I. $D''' > 0$ and $D' > 0$.
The slope at $\hat{p} = 0$ is $-D''' + 2D'$ and the slope at $\hat{p} = 1$ is $D''' - 2D'$. If $-D''' + 2D' > 0$ then there are no valid roots, whereas if $-D''' + 2D' < 0$ then roots exist if the value at $\hat{p} = \frac{1}{2}$ is negative. By Eq. (B3), the value at $\hat{p} = \frac{1}{2}$ is $\frac{3D'''+2D'}{4}$. Since $3D''' + 2D' > 0$, the value at $\hat{p} = \frac{1}{2}$ is positive and there are no valid roots to (B3).
Case II. $D''' < 0$ and $D' < 0$.
The slope at $\hat{p} = 0$ is $-D''' + 2D'$, and the value at $\hat{p} = 0$ is D''' by Eq. (B3), which is negative. Therefore, if $-D''' + 2D' < 0$ then there are no valid roots. If $-D''' + 2D' > 0$, then if the value at $\hat{p} = \frac{1}{2}$ is positive, which requires that $3D''' + 2D' > 0$, then roots exist. However, $3D''' + 2D' < 0$ because both D''' and D' are negative. Therefore, in this case there are no valid roots.
Case III. $D''' > 0$ and $D' < 0$.
If $-D''' + 2D' > 0$, there are no roots. If $-D''' + 2D' < 0$, then roots exist if $3D''' + 2D' < 0$, or $D''' < -\frac{2}{3}D'$. In Case III this is possible and therefore two roots may exist.
Case IV. $D''' < 0$ and $D' > 0$.
If $-D''' + 2D' > 0$ then roots exist if $3D''' + 2D' > 0$, or $D''' > -\frac{2}{3}D'$, which is possible in this case.

By symmetry, if one (or more) variant's frequency is zero, equilibria include $\boldsymbol{p}^* = (1, 0, 0)$, $(0, 1, 0)$, $(0, 0, 1)$, $(\frac{1}{2}, \frac{1}{2}, 0)$, $(\frac{1}{2}, 0, \frac{1}{2})$, $(0, \frac{1}{2}, \frac{1}{2})$, and $(\hat{p}, 1 - \hat{p}, 0)$, $(1 - \hat{p}, \hat{p}, 0)$, $(\hat{p}, 0, 1 - \hat{p})$, $(1 - \hat{p}, 0, \hat{p})$, $(0, \hat{p}, 1 - \hat{p})$, $(0, 1 - \hat{p}, \hat{p})$, where $\hat{p}$ is given by solutions of (B3).

Now let $0 < p_1, p_2, p_3 < 1$ and $p_2 = p_3$, so $p_2 = \frac{1}{2}(1 - p_1)$. Then from (B1a), at equilibrium,

$$\begin{aligned} 0 = {} & D'''[(2p_1 - 1)(p_1^2 - p_1 + 1) + (\tfrac{7}{8})(1 - p_1)^3] + D''[p_1^2(1 - p_1) - \tfrac{1}{4}(1 - p_1)^3] \\ & + D'[p_1^2(1 - p_1) - \tfrac{1}{2}p_1(1 - p_1)^2] + 3D[\tfrac{1}{4}p_1(1 - p_1)^2 - \tfrac{1}{8}(1 - p_1)^3], \end{aligned} \qquad \text{[B4]}$$

which can be rearranged to

$$0 = (p_1 - \tfrac{1}{3})\{D'''(\tfrac{9}{8}p_1^2 + \tfrac{3}{8}) + \tfrac{3}{4}D''(1 - p_1^2) + \tfrac{3}{2}D'(1 - p_1)p_1 + \tfrac{9}{8}D(p_1 - 1)^2\}. \qquad \text{[B5]}$$

Therefore, either $p_1 = \frac{1}{3}$, in which case $p_2 = p_3 = \frac{1}{3}$ as well, or $p_1 \neq \frac{1}{3}$, in which case

$$0 = D'''(\tfrac{9}{8}p_1^2 + \tfrac{3}{8}) + \tfrac{3}{4}D''(1 - p_1^2) + \tfrac{3}{2}D'(1 - p_1)p_1 + \tfrac{9}{8}D(p_1 - 1)^2. \qquad \text{[B6]}$$

The solutions to (B6) are

$$p_1^* = \frac{-2D' + 3D \pm \sqrt{-3D'''^2 - 4D'''(D'' - D' + 3D) + 4(D'' + D')^2}}{3D''' - 2D'' - 4D' + 3D}, \qquad \text{[B7]}$$

provided the denominator is non-zero. Both solutions (B7) may exist, and by symmetry there may be six equilibria, as illustrated in Figure 2. Note, there may be other interior equilibria where p_1, p_2, p_3 are all unequal.

The corners are locally stable if $D''' > 0$ (SI D), and the equilibria $(0, \frac{1}{2}, \frac{1}{2})$, $(\frac{1}{2}, 0, \frac{1}{2})$, and $(\frac{1}{2}, \frac{1}{2}, 0)$ are locally stable within the two-dimensional boundary if $-16 < 3D''' + 2D' < 0$ (see Result 2 in (1)). Within this boundary, it is possible that no equilibria are stable, in which case a two-generation cycle in variant frequencies arises (see Figure 2 in (1)).

For the external stability of the boundary equilibria, assume, for example, that $p_1 = \varepsilon_1$, $p_2 = p_2^* + \varepsilon_2$, and $p_3 = 1 - p_2^* - \varepsilon_1 - \varepsilon_2$, where p_2^* could equal $\frac{1}{2}$ or be a solution to (B3). Then by (B1a),

$$\varepsilon_1' = \varepsilon_1\{1 - D''' + p_2^*(1 - p_2^*)[2D'''(2 - p_2^*(1 - p_2^*)) - 2D''(1 - 2p_2^*(1 - p_2^*)) - 6Dp_2^*(1 - p_2^*)]\}. \qquad \text{[B8]}$$

If $p_2^* = \frac{1}{2}$ then

$$\varepsilon_1' = \varepsilon_1(1 - \tfrac{1}{8}D''' - \tfrac{1}{4}D'' - \tfrac{3}{8}D), \qquad \text{[B9]}$$

so the equilibria $(0, \frac{1}{2}, \frac{1}{2})$, $(\frac{1}{2}, 0, \frac{1}{2})$, and $(\frac{1}{2}, \frac{1}{2}, 0)$ are stable when $0 < \frac{1}{2}D''' + D'' + \frac{3}{2}D < 8$. Due to the upper bounds $D''' < 1$, $D'' < 2$ and $D < 3$, the quantity $\frac{1}{2}D''' + D'' + \frac{3}{2}D$ is always less than 8. Therefore, these equilibria are stable when $0 < \frac{1}{2}D''' + D'' + \frac{3}{2}D$.

For the stability of $(\frac{1}{3}, \frac{1}{3}, \frac{1}{3})$, let $p_1 = \frac{1}{3} + \varepsilon_1$, $p_2 = \frac{1}{3} + \varepsilon_2$, and $p_3 = \frac{1}{3} - \varepsilon_1 - \varepsilon_2$. Then

$$\varepsilon_i' = \varepsilon_i(1 + \tfrac{1}{9}D''' + \tfrac{4}{27}D'' + \tfrac{2}{27}D' + \tfrac{1}{9}D) \qquad i = 1, 2. \qquad \text{[B10]}$$

Therefore the equilibrium $(\frac{1}{3}, \frac{1}{3}, \frac{1}{3})$ is stable if $-2 < \frac{1}{9}D''' + \frac{4}{27}D'' + \frac{2}{27}D' + \frac{1}{9}D < 0$.

Supplementary Information C: Symmetric equilibria with n role models and m variants

By Eq. (4), there is an equilibrium $\boldsymbol{p}' = \boldsymbol{p}$ where $\boldsymbol{p} = (p_1, p_2, \ldots, p_m)$ iff

$$\sum_{\boldsymbol{x}} D_i(\boldsymbol{x}) \frac{n!}{x_1! x_2! \cdots x_m!} p_1^{x_1} p_2^{x_2} \cdots p_m^{x_m} = 0 \qquad \text{[C1]}$$

for all $i = 1, 2, \ldots, m$. For example, at the corner $\boldsymbol{p} = (1, 0, \ldots, 0)$, we have $p_i = 0$ for $i = 2, \ldots, m$ and $p_1 = 1$, and for all $i = 1, 2, \ldots, m$,

$$\sum_{\boldsymbol{x}} D_i(\boldsymbol{x}) \frac{n!}{x_1! x_2! \cdots x_m!} p_1^{x_1} p_2^{x_2} \cdots p_m^{x_m} = D_i(n, 0, \ldots, 0) = 0, \qquad \text{[C2]}$$

since $x_1 = n$, $x_i = 0$ for $i = 2, \ldots, m$ and $\boldsymbol{D}(n, 0, \ldots, 0) = (0, 0, \ldots, 0)$.

We show now that Eq. (C1) holds for $\boldsymbol{p} = (\frac{1}{m}, \frac{1}{m}, \ldots, \frac{1}{m})$. In this case, (C1) reduces to

$$\frac{n!}{m^n} \sum_{\boldsymbol{x}} D_i(\boldsymbol{x}) \frac{1}{x_1! x_2! \cdots x_m!} = 0 \qquad i = 1, 2, \ldots, m. \qquad \text{[C3]}$$

With any role model state $\boldsymbol{x} = (x_1, x_2, \ldots, x_m)$ we associate the set $G(\boldsymbol{x})$ of all states $\boldsymbol{y} = (y_1, y_2, \ldots, y_m)$ that are symmetric to $\boldsymbol{x}$, namely having the same components in all possible orders. For example with $n = 3$ and $m = 3$ let $\boldsymbol{x} = (2, 1, 0)$. Then the set $G(\boldsymbol{x})$ is

$$G(\boldsymbol{x}) = \{(2,1,0), (2,0,1), (0,2,1), (0,1,2), (1,2,0), (1,0,2)\}. \qquad \text{[C4]}$$

If $\boldsymbol{y} \in G(\boldsymbol{x})$ then $G(\boldsymbol{y}) = G(\boldsymbol{x})$, but if $\boldsymbol{y} \notin G(\boldsymbol{x})$ then $G(\boldsymbol{y}) \cap G(\boldsymbol{x})$ is empty. Thus the set G of all role models states can be represented as the union of disjoint sets $G(\boldsymbol{z})$, namely

$$G = G(\boldsymbol{z}_1) \cup G(\boldsymbol{z}_2) \cdots G(\boldsymbol{z}_k). \qquad \text{[C5]}$$

For example, with $n = 4$ and $m = 3$, the set $G(\boldsymbol{z}_1)$ could contain $\boldsymbol{z}_1 = (3, 1, 0)$ and symmetrical configurations whereas the set $G(\boldsymbol{z}_2)$ could contain $\boldsymbol{z}_2 = (2, 1, 1)$ and symmetrical configurations.

In addition, if $\boldsymbol{y} \in G(\boldsymbol{x})$ then $y_1! y_2! \cdots y_m! = x_1! x_2! \cdots x_m!$ and so by (C3),

$$\frac{n!}{m^n} \sum_{\boldsymbol{y} \in G(\boldsymbol{x})} D_i(\boldsymbol{y}) \frac{1}{y_1! y_2! \cdots y_m!} = \frac{n!}{m^n} \left(\frac{1}{x_1! x_2! \cdots x_m!} \right) \sum_{\boldsymbol{y} \in G(\boldsymbol{x})} D_i(\boldsymbol{y}). \qquad \text{[C6]}$$

Now by the definition of $\boldsymbol{D}(\boldsymbol{y}) = (D_1(\boldsymbol{y}), D_2(\boldsymbol{y}), \ldots, D_m(\boldsymbol{y}))$, we have $\sum_{i=1}^{m} D_i(\boldsymbol{y}) = 0$ for all $\boldsymbol{y} \in G(\boldsymbol{x})$ and so $\sum_{\boldsymbol{y} \in G(\boldsymbol{x})} \sum_{i=1}^{m} D_i(\boldsymbol{y}) = 0$. Therefore,

$$\sum_{\boldsymbol{y} \in G(\boldsymbol{x})} \sum_{i=1}^{m} D_i(\boldsymbol{y}) = \sum_{i=1}^{m} \sum_{\boldsymbol{y} \in G(\boldsymbol{x})} D_i(\boldsymbol{y}) = 0. \qquad \text{[C7]}$$

Because there is complete symmetry between all m variants $A_1, A_2, \ldots, A_m$ in $G(\boldsymbol{x})$, we have

$$\sum_{\boldsymbol{y} \in G(\boldsymbol{x})} D_i(\boldsymbol{y}) = \sum_{\boldsymbol{y} \in G(\boldsymbol{x})} D_j(\boldsymbol{y}) \qquad i, j = 1, 2, \ldots, m. \qquad \text{[C8]}$$

Together (C7) and (C8) imply that

$$\sum_{\boldsymbol{y} \in G(\boldsymbol{x})} D_i(\boldsymbol{y}) = 0 \qquad i = 1, 2, \ldots, m. \qquad \text{[C9]}$$

As a result, by (C5)

$$\sum_{\boldsymbol{x}} D_i(\boldsymbol{x}) = \sum_{j=1}^{k} \sum_{\boldsymbol{x} \in G(\boldsymbol{z}_j)} D_i(\boldsymbol{x}) = 0 \qquad \text{[C10]}$$

as desired.

A similar proof applies to any boundary symmetric point $\boldsymbol{p} = (\frac{1}{\ell}, \frac{1}{\ell}, \ldots, \frac{1}{\ell}, 0, \ldots, 0)$ as then

$$\sum_{\boldsymbol{x}} D_i(\boldsymbol{x}) \frac{n!}{x_1! x_2! \cdots x_m!} p_1^{x_1} p_2^{x_2} \cdots p_m^{x_m} = \sum_{\boldsymbol{x} = (x_1, x_2, \ldots, x_\ell)} D_i(\boldsymbol{x}) \frac{n!}{x_1! x_2! \cdots x_m!} \left(\frac{1}{\ell} \right)^n \qquad \text{[C11]}$$

and we can continue as before where ℓ replaces m. Hence, in the general case of n role models and m variants, symmetric points such as $(\frac{1}{m}, \ldots, \frac{1}{m})$, $(\frac{1}{m-1}, \ldots, \frac{1}{m-1}, 0)$, etc. up to $(\frac{1}{2}, \frac{1}{2}, 0, \ldots, 0)$ are equilibria of the general recursion system (4).

Supplementary Information D: Local stability of equilibria for n role models and m variants

Central polymorphic equilibrium. To check the local stability of the polymorphic equilibrium $\boldsymbol{p}^* = (\frac{1}{m}, \frac{1}{m}, \dots, \frac{1}{m})$, set $p_i = \frac{1}{m} + \varepsilon_i$ and $p_i' = \frac{1}{m} + \varepsilon_i'$ for $i = 1, 2, \dots, m$. Then from Eq. (4),

$$\tfrac{1}{m} + \varepsilon_i' = \tfrac{1}{m} + \varepsilon_i + \tfrac{1}{n} \sum_{\boldsymbol{x}} D_i(\boldsymbol{x}) \tfrac{n!}{x_1! x_2! \cdots x_m!} \prod_{j=1}^{m} \left(\tfrac{1}{m} + \varepsilon_j\right)^{x_j} \quad \text{[D1]}$$

for $i = 1, 2, \dots, m$. The local stability conditions for $\boldsymbol{p}^*$ are determined by the linear approximation of the right-hand side of Eq. (D1). Retaining linear terms in $\varepsilon_1, \varepsilon_2, \dots, \varepsilon_m$ we have

$$\left(\tfrac{1}{m} + \varepsilon_j\right)^{x_j} \simeq \left(\tfrac{1}{m}\right)^{x_j} + x_j \left(\tfrac{1}{m}\right)^{x_j - 1} \varepsilon_j = \left(\tfrac{1}{m}\right)^{x_j} (1 + m x_j \varepsilon_j). \quad \text{[D2]}$$

As $\sum_{j=1}^{m} x_j = n$, the linear approximation of Eq. (D1) is

$$\tfrac{1}{m} + \varepsilon_i' = \tfrac{1}{m} + \varepsilon_i + \tfrac{1}{n \cdot m^n} \sum_{\boldsymbol{x}} D_i(\boldsymbol{x}) \tfrac{n!}{x_1! x_2! \cdots x_m!} \left(1 + m \sum_{j=1}^{m} x_j \varepsilon_j\right) \quad \text{[D3]}$$

for $i = 1, 2, \dots, m$. Because $\boldsymbol{p}^* = (\frac{1}{m}, \frac{1}{m}, \dots, \frac{1}{m})$ is an equilibrium of (4), we know that $\sum_{\boldsymbol{x}} D_i(\boldsymbol{x}) \frac{n!}{x_1! x_2! \dots x_m!} = 0$ for all $i = 1, 2, \dots, m$ (see Eq. (C3)). Thus Eq. (D3) reduces to

$$\varepsilon_i' = \varepsilon_i + \frac{1}{n \cdot m^{n-1}} \sum_{j=1}^{m} \left[\sum_{\boldsymbol{x}} D_i(\boldsymbol{x}) \frac{n!}{x_1! x_2! \cdots x_m!} \cdot x_j \right] \varepsilon_j. \quad \text{[D4]}$$

Due to the symmetry of $\boldsymbol{D}(\boldsymbol{x}) = (D_1(\boldsymbol{x}), D_2(\boldsymbol{x}), \dots, D_m(\boldsymbol{x}))$ with respect to the m variants $A_1, A_2, \dots, A_m$ we can write for each i

$$\frac{1}{n \cdot m^{n-1}} \sum_{\boldsymbol{x}} D_i(\boldsymbol{x}) \frac{n!}{x_1! x_2! \cdots x_m!} \cdot x_i = \alpha \qquad i = 1, \dots, m \quad \text{[D5a]}$$

$$\frac{1}{n \cdot m^{n-1}} \sum_{\boldsymbol{x}} D_i(\boldsymbol{x}) \frac{n!}{x_1! x_2! \cdots x_m!} \cdot x_j = \beta \qquad i, j = 1, \dots, m, \ i \neq j \quad \text{[D5b]}$$

and Eq. (D4) becomes

$$\varepsilon_i' = \varepsilon_i + \alpha \varepsilon_i + \beta \sum_{j \neq i} \varepsilon_j \qquad i = 1, 2, \dots, m. \quad \text{[D6]}$$

But as $\sum_{j=1}^{m} \varepsilon_j = 0$, Eq. (D6) reduces to

$$\varepsilon_i' = [1 + (\alpha - \beta)] \varepsilon_i \qquad i = 1, 2, \dots, m. \quad \text{[D7]}$$

Hence, the Jacobian of the linear approximation of Eq. (4) near $\boldsymbol{p}^* = (\frac{1}{m}, \frac{1}{m}, \dots, \frac{1}{m})$ is a scalar matrix $[1 + (\alpha - \beta)] I$ with one eigenvalue, $[1 + (\alpha - \beta)]$. Thus for the polymorphic equilibrium $\boldsymbol{p}^* = (\frac{1}{m}, \frac{1}{m}, \dots, \frac{1}{m})$ to be locally stable we need $|1 + (\alpha - \beta)| < 1$.

Observe that by Eqs. (D5) for any $i = 1, 2, \dots, m$,

$$\alpha + (m-1)\beta = \frac{1}{n \cdot m^{n-1}} \sum_{\boldsymbol{x}} D_i(\boldsymbol{x}) \frac{n!}{x_1! x_2! \cdots x_m!} \sum_{j=1}^{m} x_j. \quad \text{[D8]}$$

But $\sum_{j=1}^{m} x_j = n$ and $\sum_{\boldsymbol{x}} D_i(\boldsymbol{x}) \frac{n!}{x_1! x_2! \cdots x_m!} = 0$ (by SI C) for all $i = 1, 2, \dots, m$. Therefore, $\alpha + (m-1)\beta = 0$ or $\beta = -\frac{1}{m-1}\alpha$. Hence, with α in Eq. (D5a), $\boldsymbol{p}^*$ is locally stable if

$$|1 + \tfrac{m}{m-1}\alpha| < 1. \quad \text{[D9]}$$

Corner equilibria. Consider the variant A_1, whose frequency is p_1. From Eq. (4),

$$p_1' = \sum_{\boldsymbol{x}} \left[\frac{x_1}{n} + \frac{D_1(\boldsymbol{x})}{n} \right] \frac{n!}{x_1! x_2! \cdots x_m!} p_1^{x_1} p_2^{x_2} \cdots p_m^{x_m}. \quad \text{[D10]}$$

Near fixation of A_1, let its frequency be $p_1 = 1 - \varepsilon$, where $\varepsilon > 0$. The frequencies of the other variants are $p_i = \varepsilon_i$, where $\varepsilon_i \geq 0$ and $\sum_{i=2}^{m} \varepsilon_i = \varepsilon > 0$. Eq. (D10) becomes

$$p_1' = 1 - \varepsilon' = 1 - \varepsilon + \frac{1}{n} \sum_{\boldsymbol{x}} D_1(\boldsymbol{x}) \frac{n!}{x_1! x_2! \cdots x_m!} (1 - \varepsilon)^{x_1} \varepsilon_2^{x_2} \cdots \varepsilon_m^{x_m}. \quad \text{[D11]}$$

Neglecting terms $O(\varepsilon^2)$, the remaining terms must have $x_1 = n-1$ and $x_i = 1$ for a given $i \neq 1$. Therefore, there is only one conformity coefficient $D_1(\boldsymbol{x}) = D$ by Eq. (17), where $\boldsymbol{x} = (n-1, 1, 0, 0, \ldots, 0)$ (or, by symmetry, $\boldsymbol{x} = (n-1, 0, 1, 0, \ldots, 0)$ and so on). Eq. (D11) becomes

$$1 - \varepsilon' = 1 - \varepsilon + \frac{1}{n} \sum_{i=2}^{m} D \cdot \frac{n!}{(n-1)!} \varepsilon_i, \quad \text{[D12]}$$

or

$$\varepsilon' = \varepsilon(1 - D). \quad \text{[D13]}$$

By (3a), $D_1(\boldsymbol{x}) < 1$. It follows that if $D = D_1(\boldsymbol{x}) > 0$, then $\varepsilon' < \varepsilon$ and the corner equilibrium is locally stable, whereas if $D = D_1(\boldsymbol{x}) < 0$, it is unstable. By symmetry, if one corner equilibrium is locally stable, all corners are locally stable.

Supplementary Information E: Classifying $D(x)$ for $n = 4$ or 5 role models and $m = 3$ variants

Tables S1 and S2 record possible configurations of role model states $\boldsymbol{x}$; the probability $P(A_i \mid \boldsymbol{x})$ that an offspring adopts variant A_i given $\boldsymbol{x}$, following Eqs. (17); and the probability $P(\boldsymbol{x})$ of the state $\boldsymbol{x}$, with $n = 4$ and $n = 5$, respectively.

Table S1. $n = 4$ **role models and** $m = 3$ **variants**

	Offspring probabilities $P(A_i \mid \boldsymbol{x})$			
Role model state, $\boldsymbol{x}$	A_1	A_2	A_3	$P(\boldsymbol{x})$
$(4,0,0)$	1	0	0	p_1^4
$(0,4,0)$	0	1	0	p_2^4
$(0,0,4)$	0	0	1	p_3^4
$(3,1,0)$	$\frac{3}{4}+\frac{D'}{4}$	$\frac{1}{4}-\frac{D'}{4}$	0	$4p_1^3p_2$
$(3,0,1)$	$\frac{3}{4}+\frac{D'}{4}$	0	$\frac{1}{4}-\frac{D'}{4}$	$4p_1^3p_3$
$(1,3,0)$	$\frac{1}{4}-\frac{D'}{4}$	$\frac{3}{4}+\frac{D'}{4}$	0	$4p_1p_2^3$
$(1,0,3)$	$\frac{1}{4}-\frac{D'}{4}$	0	$\frac{3}{4}+\frac{D'}{4}$	$4p_1p_3^3$
$(0,3,1)$	0	$\frac{3}{4}+\frac{D'}{4}$	$\frac{1}{4}-\frac{D'}{4}$	$4p_2^3p_3$
$(0,1,3)$	0	$\frac{1}{4}-\frac{D'}{4}$	$\frac{3}{4}+\frac{D'}{4}$	$4p_2p_3^3$
$(2,2,0)$	$\frac{1}{2}$	$\frac{1}{2}$	0	$6p_1^2p_2^2$
$(2,0,2)$	$\frac{1}{2}$	0	$\frac{1}{2}$	$6p_1^2p_3^2$
$(0,2,2)$	0	$\frac{1}{2}$	$\frac{1}{2}$	$6p_2^2p_3^2$
$(2,1,1)$	$\frac{1}{2}+\frac{D}{4}$	$\frac{1}{4}-\frac{D}{8}$	$\frac{1}{4}-\frac{D}{8}$	$12p_1^2p_2p_3$
$(1,2,1)$	$\frac{1}{4}-\frac{D}{8}$	$\frac{1}{2}+\frac{D}{4}$	$\frac{1}{4}-\frac{D}{8}$	$12p_1p_2^2p_3$
$(1,1,2)$	$\frac{1}{4}-\frac{D}{8}$	$\frac{1}{4}-\frac{D}{8}$	$\frac{1}{2}+\frac{D}{4}$	$12p_1p_2p_3^2$

In Table S1, in some cases, $\boldsymbol{x} = (3, 1, 0)$ or one of its symmetrical configurations, and in others, $\boldsymbol{x} = (2, 1, 1)$ or a symmetrical configuration. We let $\boldsymbol{D}(3, 1, 0) = (D', -D', 0)$ and $\boldsymbol{D}(2, 1, 1) = (D, -\frac{D}{2}, -\frac{D}{2})$ (and $\boldsymbol{D}(\boldsymbol{x})$ for the $\boldsymbol{x}$ with the same components but in different orders are found by applying the symmetry property).

In other words, following Eqs. (17), $\boldsymbol{D}(\boldsymbol{x}) = (g_1(\boldsymbol{x})d(\boldsymbol{x}),\ g_2(\boldsymbol{x})d(\boldsymbol{x}),\ g_3(\boldsymbol{x})d(\boldsymbol{x}))$, where $d(\boldsymbol{x})$ is a constant such as D' or D. For example, $D_2(3, 1, 0) = g_2(3, 1, 0) \cdot D$ with $g_2(3, 1, 0) = -1$.

Then, by Eq. (4) we have that

$$\begin{aligned} p_1' = p_1^4 &+ 4p_1^3p_2\left(\frac{3}{4}+\frac{D'}{4}\right) + 4p_1^3p_3\left(\frac{3}{4}+\frac{D'}{4}\right) + 12p_1^2p_2p_3\left(\frac{2}{4}+\frac{D}{4}\right) + 6p_1^2p_2^2\left(\frac{2}{4}\right) + 6p_1^2p_3^2\left(\frac{2}{4}\right) \\ &+ 12p_1p_2^2p_3\left(\frac{1}{4}-\frac{D}{8}\right) + 12p_1p_2p_3^2\left(\frac{1}{4}-\frac{D}{8}\right) + 4p_1p_2^3\left(\frac{1}{4}-\frac{D'}{4}\right) + 4p_1p_3^3\left(\frac{1}{4}-\frac{D'}{4}\right), \end{aligned} \quad \text{[E1]}$$

which is the same as Eq. (13) with $i = 1$.

Table S2. $n = 5$ **role models and** $m = 3$ **variants**

	Offspring probabilities $P(A_i \mid \boldsymbol{x})$			
Role model state, $\boldsymbol{x}$	A_1	A_2	A_3	$P(\boldsymbol{x})$
$(5, 0, 0)$	1	0	0	p_1^5
$(0, 5, 0)$	0	1	0	p_2^5
$(0, 0, 5)$	0	0	1	p_3^5
$(4, 1, 0)$	$\frac{4}{5} + \frac{D'''}{5}$	$\frac{1}{5} - \frac{D'''}{5}$	0	$5p_1^4p_2$
$(4, 0, 1)$	$\frac{4}{5} + \frac{D'''}{5}$	0	$\frac{1}{5} - \frac{D'''}{5}$	$5p_1^4p_3$
$(1, 4, 0)$	$\frac{1}{5} - \frac{D'''}{5}$	$\frac{4}{5} + \frac{D'''}{5}$	0	$5p_1p_2^4$
$(1, 0, 4)$	$\frac{1}{5} - \frac{D'''}{5}$	0	$\frac{4}{5} + \frac{D'''}{5}$	$5p_1p_3^4$
$(0, 4, 1)$	0	$\frac{4}{5} + \frac{D'''}{5}$	$\frac{1}{5} - \frac{D'''}{5}$	$5p_2^4p_3$
$(0, 1, 4)$	0	$\frac{1}{5} - \frac{D'''}{5}$	$\frac{4}{5} + \frac{D'''}{5}$	$5p_2p_3^4$
$(3, 1, 1)$	$\frac{3}{5} + \frac{D''}{5}$	$\frac{1}{5} - \frac{D''}{10}$	$\frac{1}{5} - \frac{D''}{10}$	$20p_1^3p_2p_3$
$(1, 3, 1)$	$\frac{1}{5} - \frac{D''}{10}$	$\frac{3}{5} + \frac{D''}{5}$	$\frac{1}{5} - \frac{D''}{10}$	$20p_1p_2^3p_3$
$(1, 1, 3)$	$\frac{1}{5} - \frac{D''}{10}$	$\frac{1}{5} - \frac{D''}{10}$	$\frac{3}{5} + \frac{D''}{5}$	$20p_1p_2p_3^3$
$(3, 2, 0)$	$\frac{3}{5} + \frac{D'}{5}$	$\frac{2}{5} - \frac{D'}{5}$	0	$10p_1^3p_2^2$
$(3, 0, 2)$	$\frac{3}{5} + \frac{D'}{5}$	0	$\frac{2}{5} - \frac{D'}{5}$	$10p_1^3p_3^2$
$(2, 3, 0)$	$\frac{2}{5} - \frac{D'}{5}$	$\frac{3}{5} + \frac{D'}{5}$	0	$10p_1^2p_2^3$
$(2, 0, 3)$	$\frac{2}{5} - \frac{D'}{5}$	0	$\frac{3}{5} + \frac{D'}{5}$	$10p_1^2p_3^3$
$(0, 3, 2)$	0	$\frac{3}{5} + \frac{D'}{5}$	$\frac{2}{5} - \frac{D'}{5}$	$10p_2^3p_3^2$
$(0, 2, 3)$	0	$\frac{2}{5} - \frac{D'}{5}$	$\frac{3}{5} + \frac{D'}{5}$	$10p_2^2p_3^3$
$(2, 2, 1)$	$\frac{2}{5} + \frac{D}{10}$	$\frac{2}{5} + \frac{D}{10}$	$\frac{1}{5} - \frac{D}{5}$	$30p_1^2p_2^2p_3$
$(2, 1, 2)$	$\frac{2}{5} + \frac{D}{10}$	$\frac{1}{5} - \frac{D}{5}$	$\frac{2}{5} + \frac{D}{10}$	$30p_1^2p_2p_3^2$
$(1, 2, 2)$	$\frac{1}{5} - \frac{D}{5}$	$\frac{2}{5} + \frac{D}{10}$	$\frac{2}{5} + \frac{D}{10}$	$30p_1p_2^2p_3^2$

In Table S2, there are four distinct groups of symmetrical configurations of $\boldsymbol{x}$. We let $\boldsymbol{D}(4,1,0) = (D''', -D''', 0)$, $\boldsymbol{D}(3,1,1) = (D'', -\frac{D''}{2}, -\frac{D''}{2})$, $\boldsymbol{D}(3,2,0) = (D', -D', 0)$, and $\boldsymbol{D}(2,2,1) = (\frac{D}{2}, \frac{D}{2}, -D)$. In terms of Eq. (17a), for example, $D_2(3,1,1) = g_2(3,1,1)d(3,1,1)$ where $d(3,1,1) = D''$ and $g_2(3,1,1) = -\frac{1}{2}$. Again, the $\boldsymbol{D}(\boldsymbol{x})$ for the symmetrical configurations can be found using the symmetry property. Therefore, by (4), the recursion in p_1 is

$$\begin{aligned} p_1' = p_1^5 &+ \left(\frac{4}{5} + \frac{D'''}{5}\right) 5p_1^4p_2 + \left(\frac{4}{5} + \frac{D'''}{5}\right) 5p_1^4p_3 + \left(\frac{3}{5} + \frac{D'}{5}\right) 10p_1^3p_2^2 + \left(\frac{3}{5} + \frac{D'}{5}\right) 10p_1^3p_3^2 \\ &+ \left(\frac{3}{5} + \frac{D''}{5}\right) 20p_1^3p_2p_3 + \left(\frac{2}{5} + \frac{D}{10}\right) 30p_1^2p_2^2p_3 + \left(\frac{2}{5} + \frac{D}{10}\right) 30p_1^2p_2p_3^2 + \left(\frac{2}{5} - \frac{D'}{5}\right) 10p_1^2p_2^3 \\ &+ \left(\frac{2}{5} - \frac{D'}{5}\right) 10p_1^2p_3^3 + \left(\frac{1}{5} - \frac{D''}{10}\right) 20p_1p_2^3p_3 + \left(\frac{1}{5} - \frac{D''}{10}\right) 20p_1p_2p_3^3 + \left(\frac{1}{5} - \frac{D}{5}\right) 30p_1p_2^2p_3^2 \\ &+ \left(\frac{1}{5} - \frac{D'''}{5}\right) 5p_1p_2^4 + \left(\frac{1}{5} - \frac{D'''}{5}\right) 5p_1p_3^4, \end{aligned} \quad \text{[E2]}$$

which can be rearranged to become Eq. (B1a).

Supplementary Information F: Bounds on the conformity coefficient $d(\boldsymbol{x})$

The lower bound of $d(\boldsymbol{x})$ occurs when $d(\boldsymbol{x}) < 0$, and by Eq. (17a),

$$-x_i < g_i(\boldsymbol{x})d(\boldsymbol{x}). \quad \text{[F1]}$$

To minimize the right-hand side of (F1), let $g_i(\boldsymbol{x}) > 0$, in which case $g_i(\boldsymbol{x}) = \frac{x_i}{\sum_{z \in \Pi} z}$ and

$$-\frac{x_i}{g_i(\boldsymbol{x})} = -\sum_{z \in \Pi} z < d(\boldsymbol{x}). \quad \text{[F2]}$$

To calculate the upper bound of $d(\boldsymbol{x})$, which occurs when $d(\boldsymbol{x}) > 0$, rearrange Eq. (17a) to

$$g_i(\boldsymbol{x})d(\boldsymbol{x}) < n - x_i. \quad \text{[F3]}$$

The left-hand side of (F3) is maximized when $g_i(\boldsymbol{x}) > 0$ because $d(\boldsymbol{x}) > 0$. Therefore, dividing both sides by $g_i(\boldsymbol{x}) = \frac{x_i}{\sum_{z \in \Pi} z}$ gives

$$d(\boldsymbol{x}) < \frac{n - x_i}{g_i(\boldsymbol{x})} = \sum_{z \in \Pi} z \left(\frac{n}{x_i} - 1\right), \quad \text{[F4]}$$

and the right-hand side of (F4) is minimized when $x_i = \max_{x_i \in \boldsymbol{x}} x_i$. Therefore, the bounds on $d(\boldsymbol{x})$ are

$$-\sum_{z \in \mathrm{II}} z < d(\boldsymbol{x}) < \sum_{z \in \mathrm{II}} z \left(\frac{n}{\max_{x_i \in \boldsymbol{x}} x_i} - 1 \right). \tag{F5}$$

Supplementary Information G: An alternative classification of $D(x)$

Here, we suggest an alternative classification of Eq. (17b) for $g_i(\boldsymbol{x})$, where $\boldsymbol{x} = (x_1, x_2, \ldots, x_m)$ and the offspring probabilities are

$$P(A_i \mid \boldsymbol{x}) = \frac{x_i}{n} + \frac{D_i(\boldsymbol{x})}{n} = \frac{x_i}{n} + \frac{d(\boldsymbol{x}) g_i(\boldsymbol{x})}{n} \quad i = 1, 2, \ldots, m. \tag{G1}$$

Notice that when all the components of $\boldsymbol{x}$ are equal, namely $\boldsymbol{x} = (\ell, \ell, \ldots, \ell)$, then by the symmetry $\boldsymbol{D}(\boldsymbol{x}) = (0, 0, \ldots, 0)$. If this is not the case, let

$$\begin{aligned} \mathrm{I}(\boldsymbol{x}) &= \{j : x_j = 0\} \\ \mathrm{II}(\boldsymbol{x}) &= \{j : x_j = \max_{1 \le i \le m} x_i\} \\ \mathrm{III}(\boldsymbol{x}) &= \{j : 0 < x_j < \max_{1 \le i \le m} x_i\}. \end{aligned} \tag{G2}$$

Then we suggest the following alternative to the classification in Eq. (17b) of $g_i(\boldsymbol{x})$:

$$g_i(\boldsymbol{x}) = \begin{cases} 0 & \text{if } i \in \mathrm{I}(\boldsymbol{x}) \\ \frac{x_i}{\sum_{j \in \mathrm{II}(\boldsymbol{x})} x_j} & \text{if } i \in \mathrm{II}(\boldsymbol{x}) \\ -\frac{x_i^{-1}}{\sum_{j \in \mathrm{III}(\boldsymbol{x})} x_j^{-1}} & \text{if } i \in \mathrm{III}(\boldsymbol{x}). \end{cases} \tag{G3}$$

Observe that when $i \in \mathrm{II}(\boldsymbol{x})$ then $\frac{x_i}{\sum_{j \in \mathrm{II}(\boldsymbol{x})} x_j} = \frac{1}{m'}$ where m' is the number of components x_j such that $x_j = \max_{1 \le i \le m} x_i$.

Supplementary Information H: Proof of Result 5

For convergence of Eq. (19) to an equilibrium, the following properties of $g_i(\boldsymbol{x})$ are useful:
$\mathbb{Q}$(i) Suppose $n > x_1 > x_2 > 0$. Then $g_1(\boldsymbol{x}) > g_2(\boldsymbol{x})$.
$\mathbb{Q}$(ii) If $p_1 = \max_{1 \le i \le m} p_i < 1$ and $d(\boldsymbol{x}) > 0$, then for $i = 2, 3, \ldots, m$,

$$\sum_{\boldsymbol{x}} d(\boldsymbol{x}) g_1(\boldsymbol{x}) \frac{n!}{x_1! \cdots x_m!} p_1^{x_1} \cdots p_m^{x_m} > \sum_{\boldsymbol{x}} d(\boldsymbol{x}) g_i(\boldsymbol{x}) \frac{n!}{x_1! \cdots x_m!} p_1^{x_1} \cdots p_m^{x_m}. \tag{H1}$$

To show $\mathbb{Q}$(i), note that from Eq. (17b), if $x_1 \in \mathrm{I}$ then $x_1 = \frac{n}{r}$ and $0 < x_2 < \frac{n}{r}$, implying that $g_1(\boldsymbol{x}) = 0 > g_2(\boldsymbol{x})$. If $x_1 \in \mathrm{II}$ then $\frac{n}{r} < x_1 < n$ and if $x_2 \in \mathrm{II}$ then $x_1 > x_2$ implies $g_1(\boldsymbol{x}) > g_2(\boldsymbol{x})$. If $x_2 \in \mathrm{III}$ then $g_1(\boldsymbol{x}) > 0 > g_2(\boldsymbol{x})$. Finally, if $x_1 \in \mathrm{III}$, then since $x_1 > x_2 > 0$, we have $x_2 \in \mathrm{III}$ and $x_1^{-1} < x_2^{-1}$. Hence $0 > g_1(\boldsymbol{x}) > g_2(\boldsymbol{x})$.

For $\mathbb{Q}$(ii), without loss of generality we show that if $0 < p_2 < p_1 < 1$ then

$$\sum_{\boldsymbol{x}} d(\boldsymbol{x}) [g_1(\boldsymbol{x}) - g_2(\boldsymbol{x})] \frac{n!}{x_1! \cdots x_m!} p_1^{x_1} \cdots p_m^{x_m} > 0. \tag{H2}$$

For any $\boldsymbol{x} = (x_1, x_2, x_3, \ldots, x_m)$ write $\widetilde{\boldsymbol{x}} = (x_2, x_1, x_3, \ldots, x_m)$, which has the same components as $\boldsymbol{x}$ but in a different order, and therefore $d(\boldsymbol{x}) = d(\widetilde{\boldsymbol{x}})$. Consider the two terms

$$d(\boldsymbol{x}) [g_1(\boldsymbol{x}) - g_2(\boldsymbol{x})] \frac{n!}{x_1! \cdots x_m!} p_1^{x_1} p_2^{x_2} \cdots p_m^{x_m} \tag{H3a}$$

$$d(\boldsymbol{x}) [g_1(\widetilde{\boldsymbol{x}}) - g_2(\widetilde{\boldsymbol{x}})] \frac{n!}{x_1! \cdots x_m!} p_1^{x_2} p_2^{x_1} \cdots p_m^{x_m}. \tag{H3b}$$

Due to symmetry, $g_1(\widetilde{\boldsymbol{x}}) = g_2(\boldsymbol{x})$ and $g_2(\widetilde{\boldsymbol{x}}) = g_1(\boldsymbol{x})$. Hence, $d(\boldsymbol{x})[g_1(\boldsymbol{x}) - g_2(\boldsymbol{x})] = -d(\boldsymbol{x})[g_1(\widetilde{\boldsymbol{x}}) - g_2(\widetilde{\boldsymbol{x}})]$ so that one of them is positive and the other is negative if $0 < x_1, x_2 < n$. The difference in the multinomial terms depends on the difference $p_1^{x_1} p_2^{x_2} - p_1^{x_2} p_2^{x_1}$. If $x_1 > x_2$,

$$p_1^{x_1} p_2^{x_2} - p_1^{x_2} p_2^{x_1} = (p_1 p_2)^{x_2} (p_1^{x_1 - x_2} - p_2^{x_1 - x_2}). \tag{H4}$$

Then as $p_1 > p_2$, the right-hand side of Eq. (H4) is positive. If $x_1 < x_2$, the quantity $p_1^{x_1} p_2^{x_2} - p_1^{x_2} p_2^{x_1}$ is negative. In addition, $|p_1^{x_1} p_2^{x_2} - p_1^{x_2} p_2^{x_1}|$ is greater when $x_1 > x_2$ than when $x_1 < x_2$ since $p_1 > p_2$, so inequality (H2) holds as desired.

The following properties of the transformation (19) are useful:

$\mathscr{R}$(i) $p_1' = p_1 + \frac{1}{n}\sum_{\boldsymbol{x}} d(\boldsymbol{x})g_1(\boldsymbol{x})\frac{n!}{x_1!x_2!\cdots x_m!}p_1^{x_1}p_2^{x_2}\cdots p_m^{x_m}$
$= p_1 + \frac{1}{n}E[d(\boldsymbol{x})g_1(\boldsymbol{x})]$

$\mathscr{R}$(ii) $p_1' - p_i' = p_1 - p_i + \frac{1}{n}E\{d(\boldsymbol{x})[g_1(\boldsymbol{x}) - g_i(\boldsymbol{x})]\}$
for $i = 2, 3, \ldots, m$

$\mathscr{R}$(iii) $E\{d(\boldsymbol{x})[g_1(\boldsymbol{x}) - g_i(\boldsymbol{x})]\}$ has a factor $(p_1 - p_i)$

$\mathscr{R}$(iv) If $p_1 = \max\limits_{1\le i\le m} p_i < 1$, then $E[d(\boldsymbol{x})g_1(\boldsymbol{x})] > 0$ because $E[d(\boldsymbol{x})g_1(\boldsymbol{x})] > E[d(\boldsymbol{x})g_i(\boldsymbol{x})]$ for all $i = 2, 3, \ldots, m$ and $\sum_{i=1}^{m} d(\boldsymbol{x})g_i(\boldsymbol{x}) = 0$ for all $\boldsymbol{x}$.

We can then prove Result 5.

Let $\boldsymbol{p}^{(t)} = (p_1^{(t)}, p_2^{(t)}, \ldots, p_m^{(t)})$ for $t = 0, 1, 2, \ldots$ be the population state at generation t. Suppose without loss of generality that $p_1^{(0)} = \max\limits_{1\le i\le m} p_i^{(0)} < 1$, that $p_2 < p_1$, and that $p_i \le p_1$ for $i = 3, \ldots, m$. Then if $d(\boldsymbol{x}) > 0$ for all $\boldsymbol{x}$, by properties $\mathscr{R}$(i)-(iv), $p_1^{(t)} = \max\limits_{1\le i\le m} p_i^{(t)}$, $p_1^{(t+1)} > p_1^{(t)}$, and $p_1^{(t+1)} - p_2^{(t+1)} > p_1^{(t)} - p_2^{(t)}$. Thus the sequences $\{p_1^{(t)}\}_{t=0}^{\infty}$ and $\{p_1^{(t)} - p_2^{(t)}\}_{t=0}^{\infty}$ are monotone increasing and bounded, implying that $\boldsymbol{p}^{(t)} = (p_1^{(t)}, p_2^{(t)}, \ldots, p_m^{(t)}) \xrightarrow[t\to\infty]{} \boldsymbol{p}^{(\infty)}$ where $\boldsymbol{p}^{(\infty)}$ is an equilibrium. (The same argument holds if p_2 is replaced by any p_j where $p_1 > p_j$.)

If p_1 is the unique maximum, i.e., $p_1 > p_i$ for $i = 2, \ldots, m$, then $\{p_1^{(t)} - p_i^{(t)}\}_{t=0}^{\infty}$ for $i = 2, \ldots, m$ are monotone increasing and bounded such that the final equilibrium $\boldsymbol{p}^{(\infty)} = (1, 0, \ldots, 0)$. If instead, the frequencies of $\ell > 1$ variants are initially equal to the maximum, namely $\max\limits_{1\le i\le m} p_i^{(0)}$, then at equilibrium, the frequencies of these variants are $\frac{1}{\ell}$ whereas the frequencies of other variants are zero. In the simplex, the surface along which this convergence occurs has measure zero.

References

1. KK Denton, Y Ram, U Liberman, MW Feldman, Cultural evolution of conformity and anticonformity. *Proc. Natl. Acad. Sci.* **117**, 13603–13614 (2020).
